# A Model of Protein Association Based on Their Hydrophobic and Electric Interactions

**DOI:** 10.1371/journal.pone.0110352

**Published:** 2014-10-17

**Authors:** Angel Mozo-Villarías, Juan Cedano, Enrique Querol

**Affiliations:** 1 Institut de Recerca Biomèdica de Lleida & Departament de Medicina Experimental, Universitat de Lleida, Lleida, Spain; 2 Laboratorio de Inmunología, Universidad de la República Regional Norte-Salto, Salto, Uruguay; 3 Institut de Biotecnologia i Biomedicina & Departament de Bioquímica i Biologia Molecular, Universitat Autònoma de Barcelona, Barcelona, Spain; Universitat Pompeu Fabra, Barcelona Research Park of Biomedicine (PRBB), Spain

## Abstract

The propensity of many proteins to oligomerize and associate to form complex structures from their constituent monomers, is analyzed in terms of their hydrophobic (**H**), and electric pseudo-dipole (**D**) moment vectors. In both cases these vectors are defined as the product of the distance between their positive and negative centroids, times the total hydrophobicity or total positive charge of the protein. Changes in the magnitudes and directions of **H** and **D** are studied as monomers associate to form larger complexes. We use these descriptors to study similarities and differences in two groups of associations: a) open associations such as polymers with an undefined number of monomers (i.e. actin polymerization, amyloid and HIV capsid assemblies); b) closed symmetrical associations of finite size, like spherical virus capsids and protein cages. The tendency of the hydrophobic moments of the monomers in an association is to align in parallel arrangements following a pattern similar to those of phospholipids in a membrane. Conversely, electric dipole moments of monomers tend to align in antiparallel associations. The final conformation of a given assembly is a fine-tuned combination of these forces, limited by steric constraints. This determines whether the association will be open (indetermined number of monomers) or closed (fixed number of monomers). Any kinetic, binding or molecular peculiarities that characterize a protein assembly, comply with the vector rules laid down in this paper. These findings are also independent of protein size and shape.

## Introduction

One of the most fundamental aspects of the knowledge of protein function is their ability to self-associate, constituting larger structures suitable for many cell structures and functions. All those characteristics involved when certain proteins form any kind of association (from dimers to sizeable oligomers) have been of great interest from the earliest moments of protein research and continue to be the object of intense research in many areas [1]–[5]. This interest covers the most basic knowledge in cell function from actin and tubulin polymerization, to those mechanisms that provoke serious diseases like Alzheimer’s, which imply large amyloid aggregations in the cytoskeleton, just to mention two conspicuous examples. These are both associative processes that are essential for cell life [6]–[9] or bring disease and death [10]–[13]. These processes may show different association kinetics among them but both may share common features that may yield some clues about necessary conditions for association. Which of these conditions are being shared by associations that end up with a definitive number of monomers, such as virus capsids, or protein cages (just to mention a few examples)? One of the fundamental questions is what can be common to all associative processes and what makes them different. The problem that thus arises is how to characterize their analogies and differences in terms of a single model. Many studies have addressed the problem from many points of view. Recently, some approaches have been developed by considering electric and hydrophobic interactions [14] but so far no unified view has been established because the intrinsic complexity of each particular process prevents or hinders the generation of a unified description, let alone predictive models. One of the limiting factors added to these difficulties lays in the dependence on the availability of complete 3D structures. The Protein Data Bank continuously increases the number of structures available, as well as their completeness and accuracy. This article is an attempt to find and use simple descriptors suitable in all protein assembly processes.

We use some of the systems mentioned above as examples for application of these descriptors. For clarity and space reasons, we cannot address all quaternary assemblies, although some will be mentioned as suitable for the same analysis. Results will be shown in two groups: a) homogeneous associations in which there are an unlimited number of monomers, and b) homogeneous systems composed of a fixed number of monomers. In the first group, actin polymerization, amyloid assembly and HIV capsid helical assemblies will be described. In the second group, we have chosen Brome Mosaic virus capsid, protein cages and Ebola membrane associated virus capsid. Our purpose is to try to find common clues in these assemblies that may be extendable to other systems of proteins. In Supporting Information other systems are analyzed and tested with this methodology.

Based on studies of hydrophobic and electrical interactions as being two of the most conspicuous forces characterizing proteins, our group has been studying protein thermostability in the past [15]–[17]. Hydrophobicity has been known to be an essential force in configuring macromolecules ever since the very first studies, but it has not been easy to formalize a theoretical system like the electric forces. Even the use of the term “hydrophobic force” could be questionable since hydrophobicity is of entropic origin. But in spite of it, empirical formalizations of hydrophobic interactions have been developed, especially by the use of force fields, as alternative to surface-area models [18], [19]. Besides a pseudo electric dipole moment vector [16], **D**, this article includes a descriptor for the hydrophobic effect, the hydrophobic moment vector (**H**), defined in a similar manner as **D** relative to the hydrophobicity of each amino acid. It will be shown how the relative magnitudes and orientations of the **D** and **H** vectors and their variations are able to describe and predict the behavior of monomers in their ability to assemble. In this description, a given protein is associated to a set of vectors, **D** and **H**, allowing the prediction of its behavior interacting with other protein vector sets. The main differences in the relative orientation of these vectors, for different types of protein associations, will be shown.

In consonance with our previous work [16]–[17], we interpret a decrease in the modulus of **D**, when two monomers form a dimer, as a favorable configuration under the electric point of view, since it means that the electric centroids are closer. As far as changes in the modulus of the hydrophobic moment **H** are concerned, the interpretation is not that immediate for the reasons given above about the nature of the hydrophobic effect. We empirically use, as a reference, the constitution of a membrane as a model of interaction and stability in terms of hydrophobic moments as is discussed in the paper. The attractiveness of this method lies in its simplicity. Its description and predictive possibilities are discussed.

As already mentioned, this method takes into account only electrical and hydrophobic interactions, leaving others, like hydrogen bonding, out of the scope for simplicity. The results presented are sufficiently significant, even though we are conscious of the importance of other interactions. The purpose is to produce a model as simple as possible.

## Results

### Transmembrane Proteins

**Figure 1:**
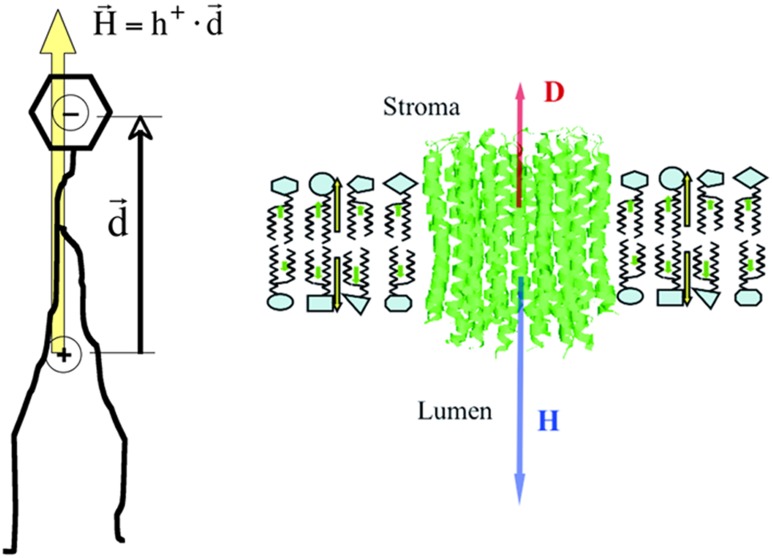
Membrane model of hydrophobic moments. Left: Cartoon representation of the hydrophobic moment vector, **H** (yellow arrow) formed in a single phospholipid within a membrane. Its modulus is defined as the product of the total hydrophobicity of the phospholipid tail, times the distance between the hydrophobic centroid (somewhere in the tail) and the hydrophilic centroid (in the polar head). Right: Schematic representation of the transmembrane protein Chloroplast ATP synthase c-ring (PDBid 3V3C) inserted in a membrane. Blue and red arrows represent hydrophobic (**H**) and electric dipole moment (**D**) vectors respectively. Small yellow arrows represent the hydrophobic moment of each layer constituting the membrane. All hydrophobic moments of the phospholipids are quasi-parallel and perpendicular to the plane of the membrane.

In order to find an interpretation of protein hydrophobic moments we studied the behavior and disposition of **H** vectors of phospholipids within a membrane and their interaction with **H** vectors of transmebrane proteins. (Vector quantities are denoted by bold characters in this article). Each phospholipid constituting a membrane has a hydrophobic centroid in its hydrophobic tail and a hydrophilic centroid in its polar head. This defines a hydrophobic moment vector approximately perpendicular to the membrane surface (see Figure 1a). The parallel alignment of phospholipids in a membrane determines the parallel alignment of their hydrophobic moments in the most energetically stable configuration. In order to compare the orientation of hydrophobic moment vectors of phospholipids in a membrane with those of transmembrane proteins, we computed the **H** and **D** vectors for a set of 40 TM proteins as well as the angles they form. Several remarkable features are worth observing.

First, for all the proteins used, their **H** vectors were always perpendicular to the membrane plane (thus, parallel to the phospholipids hydrophobic moments), whether they were directed towards the interior or the exterior of the membrane. In principle, this supports the idea that hydrophobic vectors tend to align in parallel arrangements in stable situations.

Second, it must be noted, that no single layer of phospholipids can stay single in a stable situation. As a stable membrane is composed of two opposing layers of phospholipids, hydrophobic moments of both layers tend to cancel each other out.

Third, regarding transmembrane proteins, two clearly different populations of angles between the **H** and **D** vectors (written as **D**∧**H** in the rest of the article) appeared, regardless of whether the TM proteins were of alpha or beta type. Those angles were either small (17.2°±2.3°) or large (149.1°±4.9°), indicating that the electric dipole moments tend to align with the hydrophobic moments either in a parallel or antiparallel arrangement. Figure 1b depicts a schematic example of a transmembrane all alpha protein, 3V3C (chloroplast ATP synthase), typically showing the **H** and **D** vectors perpendicular to the membrane plane.

### Protein Assemblies with an Indetermined Number of Monomers

#### Actin polymerization

Ever since the publication of the first actin structure [20], there has been a wealth of increasingly refined structures of this protein, as well as its association with other proteins. Recently, actin polymers either associated with myosin molecules or with cofilin, have been crystallized (PDBid: 1M8Q [21] and 3J0S [22] respectively), allowing a more detailed view of the configuration of the actin monomers within the polymer. We have used these structures to study actin polymerization in terms of the **H** and **D** vectors, and the angles that they form in the assembled species. These values are shown in Table S1, for both monomeric actin (G-actin) and polymeric actin (F-actin). In this table, data has been divided into several series, following the notation given in the original sources [21]–[22].

From Table S1, we computed average values for D, H and the angles that they form (written as **D**∧**H** in the rest of the article) for the individual actin monomers within the F-actin structures: <D> = 24.3±0.2 debyes; <H> = 142.4±4.8 rhu; <D∧H> = 88.3°±1.4°. Unfortunately, it is not possible to make a good comparison of these figures with values obtained from free G-actin in solution. Structures of G-actin that either come as free G-actin (1J6Z, 3HBT) or bound to other proteins, lack important segments of their sequences. For example, some structures lack the negatively charged DEDE sequence of the N-terminus. In spite of the possible variability of these magnitudes in these structures, some averages were attempted for comparison with the above averages: <D> = 25.3±5.9 debyes; <H> = 156.4±14.8 rhu and <D∧H> = 72.3°±19.3°. From these figures it can be seen that within the polymer, monomers have their **D** and **H** moments almost perpendicular to each other with little dispersion, whereas in free G-actins **D** and **H** vectors show a great variety in their relative orientations. The variability in the D∧H angle was particularly striking because values ranging from 35° to 140° were found for different structures. Another cause for dispersion in those values is that the interaction of the G-actin with its particular associate may provoke different changes in the structure of the G-actin.

**Figure 2:**
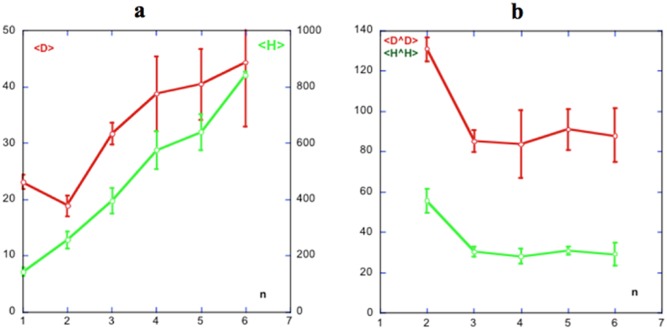
Actin polymerization. Variation of the moduli of the hydrophobic, (**H**, green) and electric dipole, (**D**, red) moment vectors as new monomers are added to the polymer. A) Variation of the moduli of both D (left vertical axis) and H (right vertical axis) with number of monomers within the polymer, n. The initial dimer formation suggests a decrease in the value of D as compared to those of the monomers. B) Variation of the angles formed by the hydrophobic, H∧H and dipole moments, D∧D, formed by the polymer and the new monomer. Note that in both cases the value of the angles decrease to steady state values. In the hydrophobic case this is a little above 20°, whereas in the dipole case, it lies between 80° and 90°, implying virtually no electric interaction. D values in debyes. H values in rhu (see methods). Error bars are from averages using the different series mentioned in the text.

When polymerizing, the moduli of both **H** and **D** vectors increase as new monomers incorporate onto the polymer, as expected given their tendency to align. There seems to be an initial decrease of D upon dimerization, followed by increasing values (Figure 2A). According to the results shown in Table S1, when an actin dimer is formed, their **H** vectors form an angle that ranges from about 40° to 70°. As a new monomer is added to the polymer, the average angle between its **D** vector and that of the assembled polymer tend to decrease to a plateau value around 30°, as seen in Figure 2B.

**Figure 3:**
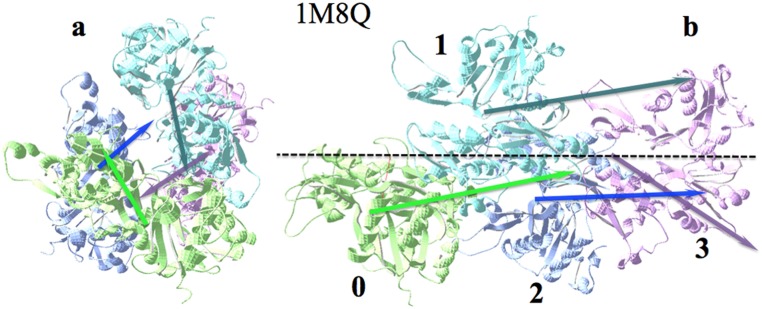
Actin polymerization. First four actin monomers from PDBid: 1M8Q, numbered from 0 to 3, following the notation given in [21]. a) View along the polymer axis: arrows are **D** vectors (colors of the arrows correspond with their actin subunits). Note the rotating pattern of these vectors as more monomers are added. b) Side view of the polymer. Hydrophobic moments keep a relatively small angle around the elongation axis.

The angles corresponding to the **H** vectors show a similar decreasing tendency from an average angle around 130° down to around 90°, as shown in Figure 2B. Figure 3a shows the cone of **H** vectors, as new actin monomers are added to the polymer. In Figure 3b it is possible to appreciate the slowly rotating pattern shown by the **D** vectors as the polymerization proceeds.

#### Amyloid formation of Aß_9–40_ peptides

**Figure 4:**
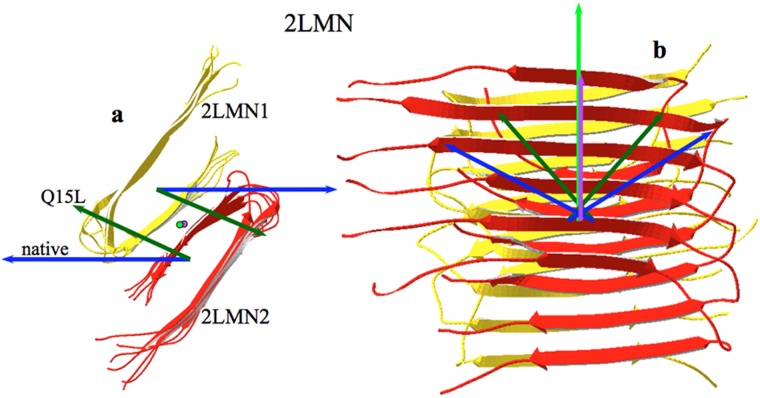
Amyloid association. Representation of two groups of six amyloid Aß_1–40_ peptides, each obtained from PDBid: 2LMN [26]. a) Profile view of the two sets (2LMN1 and 2LMN2, see text). The two dark blue arrows represent the sum of the individual **H** vectors of the respective set. The two dark green arrows represent the sum of the individual **H** vectors of the mutated sets (Q15L). In this view, the total hydrophobic moment is near zero in both native and mutated species. b) The same complexes rotated 90° towards the reader. In this position, the hydrophobic moments of both sets of native (2LMN, blue) and mutated (Q15L, green) add to those represented vertically by both the purple and light green arrows in the direction of growth. For clarity, dipole moments have not been drawn since they are very small and directed almost vertically, making the total **D** in the same direction as **H**.

A recent study by Kim and Hecht [23] revealed how peptides Aß_9–40_ (**DAEFRHDSGY EVHHQKLVFF AEDVGSNKGA IIGLMVGGVV**; PDBid: 2LMN) form aggregated structures and how these structures are modified by some mutations. These peptides have the shape of a pin [23], [24] and have a tendency to associate in a quasi-lateral manner, forming long arrays of pins. Sets of these arrays tend to associate among themselves in an antiparallel relative orientation, as shown in Figure 4.

We analyzed the behavior of the hydrophobic and electric dipole moments of these structures and results are summarized in Table S2 using the coordinates of the 2LMN crystal. These coordinates correspond to an array of six peptides (2LMN1), together with another array of six peptides (2LMN2) disposed in an antiparallel arrangement, similar to that observed in Figure 4.

Several features are worth mentioning from Table S2. It is important to observe that the association of subunits from “a” to “f” of the array 2LMN1 is such that the **H** vectors tend to align themselves in a quasi-parallel manner to each other. The same can be observed for the association of array 2LMN2 (“g” to “l”). The association of both arrays is done in such a way that their hydrophobic moments lay quasi-perpendicular (about 108°) to each other, yielding a larger total hydrophobic moment, as expected.

Under the electric point of view, the association of the peptides within an array is not favorable since the electric dipole moments are arranged parallel to each other. This fact may be taken as an example of the pre-eminence of hydrophobic effect overcoming the electric force. Nevertheless, it must be noted that the way in which both arrays 2LMN1 and 2LMN2 are associated to form 2LMN is favorable under the electrical point of view, since the total dipole moment is smaller than those of the two arrays, without challenging the hydrophobic association between them. The angle formed by the dipole moments of each array is about 145°, indicating a quasi-antiparallel arrangement.

The advantage in choosing this structure for analysis lays in the fact that Kim and Hecht [23] produced a structure in which Glutamine 15 was mutated into Leucine, that is, to a more hydrophobic species. The same analysis with the mutated complexes yielded a significantly different result from the wild type.

Since the mutation does not involve changes in electric charges, electric dipoles did not show significant changes in intensity or orientation. The hydrophobic moments intensities of the individual pin-like peptides did not change significantly from those values observed in the native species either. Consequently, the hydrophobic moments of the individual arrays of peptides are essentially the same as in the wild type peptides. However, the difference lies in the relative orientation of these hydrophobic moments: The relative angle between hydrophobic moments in the wild type was found around 108°, while in the Q15L mutant it was around 128°. This separation of both moments implies a better antiparallel alignment between both vectors and a smaller total hydrophobic moment of the whole complex. Figure 4 shows the hydrophobic moments of both arrays for the wild type and the Q15L mutant.

#### Superoxide Dismutase (SOD1)

Superoxide Dismutase responsible for Familial Amyotrophic Lateral Sclerosis is another example of a degenerative disease-related self-assembling process. The function of this protein is Cu^2+^ and Zn^2+^-dependent. Seetharaman et al. [25] describe the influence of the absence of these metal ions as well as the G93A mutation in its assembling capacity.

**Figure 5:**
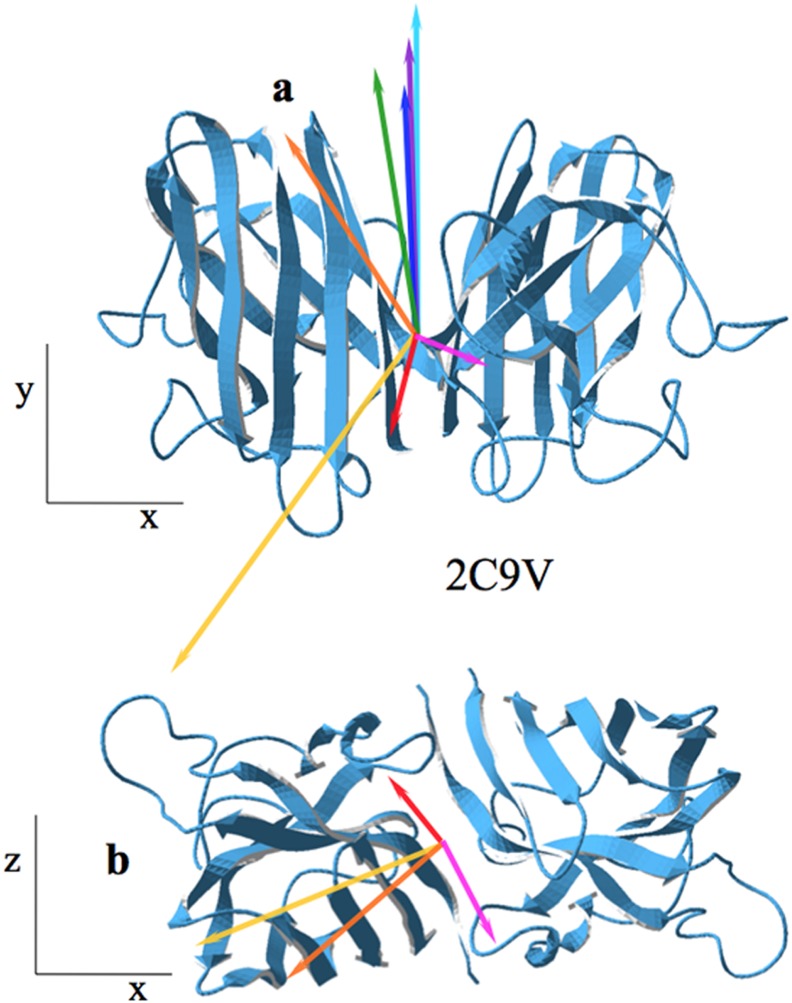
SOD1 assembly. Two views of the SOD1 dimer: a) front plane; b) rotated 90° towards the viewer. Red (**D**) and green (**H**): native dimer, PDBid: 2C9V. Magenta (**D**) and purple (**H**): depletion of metal ions (PDBid: 3ECU). Orange (**D**) and dark blue (**H**): G93A mutation (PDBid: 3GZO). Yellow (**D**) and pale blue (**H**): both depletion of metal ions plus G93A mutation (PDBid: 3GZP). For clarity, in b) hydrophobic vectors have not been drawn.

Analyzing the crystal structure of SOD1 (PDBid: 2C9V), it was found that native monomers associate as a dimer by opposing both their electric dipole (163°) and hydrophobic moments (162°). The association leaves a component of the total hydrophobic moment perpendicular to the z–y plane of the compound (green in Figure 5a), as well as a dipole moment (red in Figure 5a, b).

In this condition SOD1 does not seem to aggregate or does not do it in a virulent way. However, the absence of metal ions produces a slight deviation of the hydrophobic moment vector plus the appearance of a strong component of the dipole moment perpendicular to both the y-x and z-x planes of the dimer (magenta in Figure 5a, b), suggesting an increase of the lateral electric attractiveness towards other monomers.

Mutation G93A (PDBid: 3GZO) slightly increases the modulus of **H** but not its direction. The dipole moment vector **D**, changes direction with conspicuous components in both frontal and lateral directions, implying a strong interaction with other dimers. Both mutation plus metal ions suppression (PDBid: 3GZP), do not show a significant effect on **H**, but **D** produces significant changes in both horizontal and vertical components, suggesting a strong enhancement of its aggregability in these directions.

#### Human Immunodeficiency Virus-1 (HIV-1) capsid

**Figure 6:**
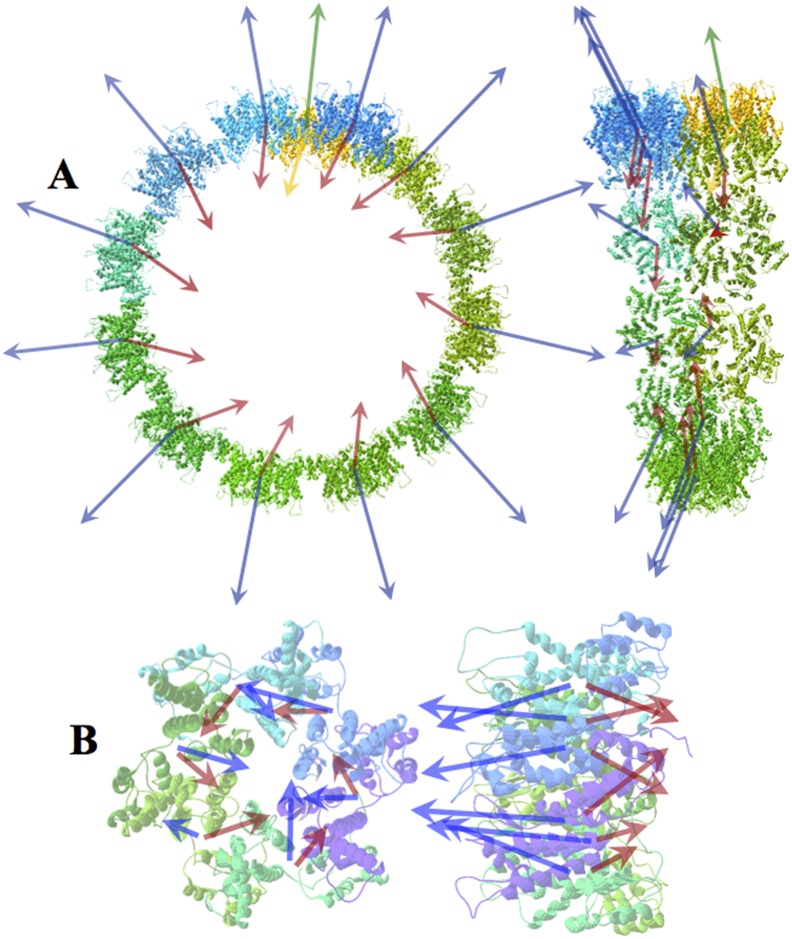
HIV capsid tubule assembly. a) Front and side views of one turn of HIV capsid (PDBid: 3J4F). Blue and red arrows represent the individual hydrophobic and dipole moments of each of the 12 hexamers in each turn of the microtubule. Green and yellow arrows are for the first hexamer of the next turn. In the side view (right), the slight leaning of the vectors toward the axis on the ensemble can be appreciated. b) Front and side views of one of the hexamers. Components of the **H** and **D** vectors of each of the six monomers within the hexamer are not symmetrical and are the cause of the deviation of both **H** and **D** vectors of the hexamer in respect to its own axis. As new hexamers are incorporated into the complex, the resulting **H_tot_** vector describes a helical trajectory around the axis of the tubule.

Zhao et al. [26] have recently published the coordinates of a HIV-1 capsid assembly (PDBid: 3J4F). The structure is a helically growing microtubule of 12 units per turn. Figure 6a shows the front and side views of the first turn where the 12 units of the turn plus the first unit of the next turn can be seen. The basic building unit of this compound is a hexamer whose subunits are arranged in the shape of a star. These hexamers assemble laterally, presumably through interactions of their respective hydrophobic moments (see Figure 6b). It is worth it to observe here the subtle combination of electric and hydrophobic effects: as new elements are added to the compound the hydrophobic interaction becomes less effective (H vectors less aligned) while at the same time this is balanced by an increase of electric attraction since angles D∧D go from parallel (31.1°) to antiparallel orientation (152.9°, see Table S3).

The **H** and **D** vectors moments of each of the six monomers in each hexamer, are aligned relatively parallel to each other and in a quasi-perpendicular direction to the plane of the hexamer (Figure 6b). The result is that the total **H** vector of the hexamer deviates some 18°–20° from its axis. On the other hand, the relative orientations of the **D** vectors are not strictly parallel but form angles of about 45° relative to each other, making their interaction not as unfavorable as if the were perfectly parallel. This also results in a total **D** vector in the hexamer not aligned with axis showing a deviation angle of about 15°.

Once the hexamers are incorporated into the turn, the **H** vectors lean over one side of the turn, projecting their components near the axis. As the tubule grows adding new turns, the total **H** vector increases in the direction of the axis of the tubule. On the other hand, the components of the **H** vectors in the plane of the turn cancel out. The way in which the individual **D** vectors of the hexamers are oriented makes the total modulus D smaller than those of the individual hexamers, which is an indication of stability, although with a net increase as the tubule grows (see Table S3).

### Protein Assemblies of Limited Numbers of Monomers

Most quaternary assemblages (homo-oligomers) of proteins are formed with a determined number of monomers in order to be biologically functional. Contrary to the cases shown in the former section, these structures do not grow indefinetively, but form structures of a certain complexity in stable configurations. The number of cases provided by the Protein Data Bank is increasingly large, so only some examples of systems of different sizes and shapes are described here as an application of this vector analysis.

#### Brome Mosaic Virus

Many virus capsids share the common feature of being closed spherical structures. In such cases the constituent monomers arrange in such a symmetric way that both the hydrophobic and electric dipole moments of the assembly are very small or zero, providing sound stability to the complex.

**Figure 7:**
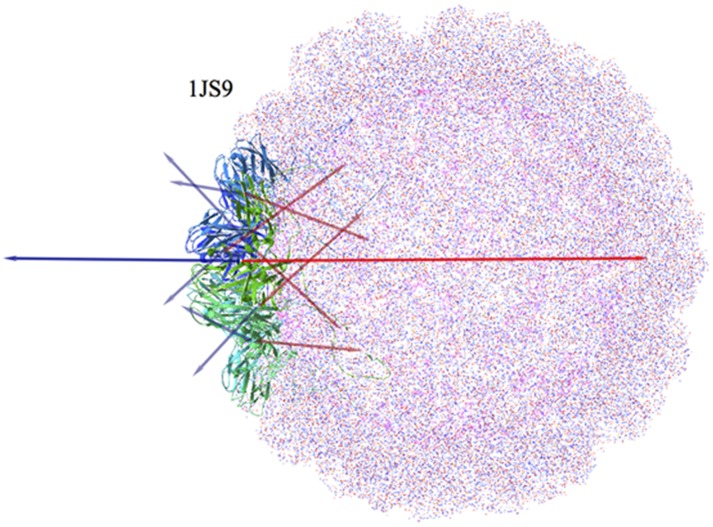
Whole Brome Mosaic Virus capsid. One of the lateral pentamers has been drawn as a ribbon and colored. Blue and red arrows represent the individual **H** and **D** vectors respectively of each of the five components constituting the pentamer. Projections of the five individual **H**_i_ vectors on the plane of the pentamer cancel each other out, whereas projections on the pentamer axis leave a net **H** vector radiating away from the centre of the capsid (long horizontal green arrow). Analogously, there is a net **D** vector pointing to the centre.

The structure of Brome Mosaic Virus (PDBid: 1JS9) is formed by 12 pentamers in a quasi-spherical configuration [27]. The “basket” disposition of the five **H** and **D** vectors of each pentamer allows on one hand, to symmetrically radiate their components in the plane of the pentamer, presumably facilitating the interaction with neighbor pentamers. On the other hand, components of both **D** and **H** in the plane perpendicular to the pentamer oriented towards the centre of the structure cancel out with those oriented in the opposite side of the sphere, as shown in Figure 7. This is possible due to the precise symmetry of this complex, contrary to what was seen in the case of the Human immunodeficiency virus-1 (HIV-1) capsid (PDBid: 3J4F), where the assembly of the basic hexamer was not “closed” due to some variability in the orientation of the **H** vectors. In the present case all the pentameric subunits point exactly to the centre of the sphere, providing spherical symmetry.

#### Octahedral Cage protein

**Figure 8:**
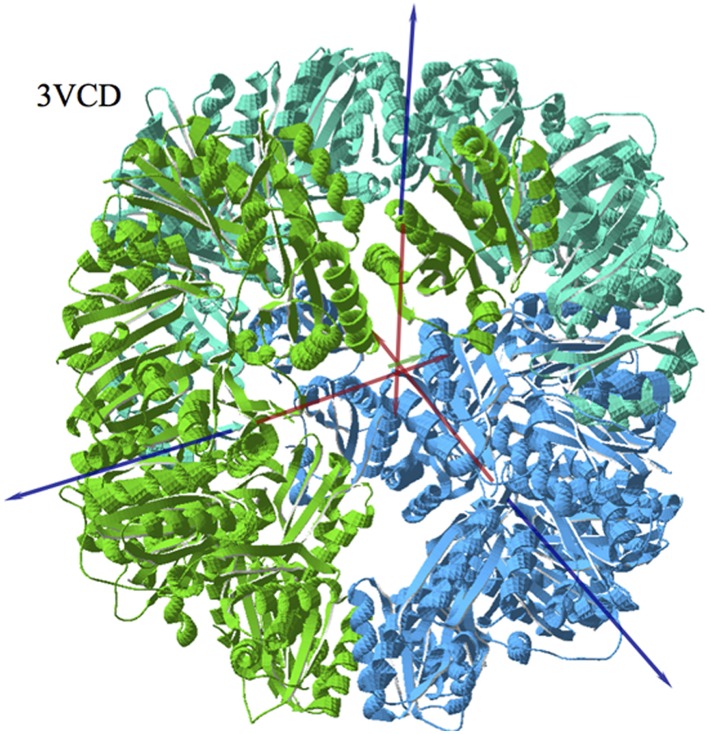
Protein cage from PDBid: 3VCD, as viewed perpendicularly to the plane defined by the electric dipole moment vectors (red arrows) and the hydrophobic moment vectors (blue arrows) of the three octamers. In this plane the total H and the total D are negligible due to the almost perfect 120° rotation symmetry.

A very promising field in Biotechnology is the design and assembling of closed protein cages. One of these assemblies is that designed by King et al. [28], the Octahedral Cage protein O333 (PDBid: 3VCD). This cage is a 24-mer formed as a trimer of octamers and each octamer is, in its turn a dimer of tetramers. They are shown in Figure 8.

We have computed the **H** and **D** vectors for each monomer, for each octamer and for the whole assembly. In Figure 8 we have superimposed the vectors corresponding to the three octamers and the whole assembly. Several points need to be described. First, for each of the three octamers, vectors **H** and **D** lay antiparallel practically in the same direction (∼171°), with the **D** vectors oriented towards the interior of the cage. Most remarkable in the whole complex, is the fact that all **H** and **D** vectors lie almost in the same equatorial plane to the whole complex. The **D** vectors are oriented at 120° with each other. This determines that the resulting **D** vector is practically zero as compared to those of the octamers. The **H** vectors are oriented ∼118° between them, providing a quasi-zero total hydrophobic vector like the case of the dipole moment. The whole ensemble thus presents 120° rotation symmetry viewed from the perpendicular to the plane of the vectors. There is a clear tendency of both **H** and **D** vectors towards small or very small values as compared to those of their components.

It should also be noted that in each octamer the **H** vector is the sum of the quasi-aligned **H** vectors of the monomers whereas the **D** vector results from the poorly aligned **D** vectors of the monomers.

Another example of a designed protein cage is that of Cu-adduct of human Ferritin (PDBid: 4DYX) by Huard et al. [30] (see description in Supporting Information).

#### Ebola virus matrix protein VP40 N-terminal domain

**Figure 9:**
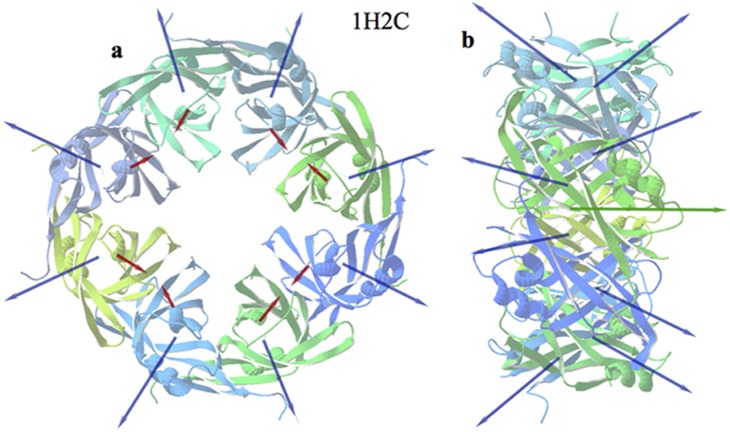
Ebola virus capsid assembly. Front (a) and side (b) views of the eight monomers that compose the Ebola virus matrix protein VP40. Blue arrows represent the individual **H** vectors of the ensemble. Red arrows are the individual **D** vectors. The green arrow in the centre is the net **H** vector. The net **D** vector is virtually zero.

This membrane-associated complex (PDBid: 1H2C) is the structural constituent of the Ebola virion [29], facilitating virus budding and it comprises a ring of eight monomers, each interacting with RNA. The peculiarity of this assembly is that it is built in such a way that the projections of the **H** and **D** vectors on the axis of the ring, point alternatively in opposite directions, leaving a residual component on the axis of the ring. The components of the **H** vectors on the plane of the ring radiate outwards with no net resultant in this plane. The alternating (antiparallel) disposition of the individual electric dipole moments confers sound stability to this complex (Figure 9).

#### Additional examples

More examples can be found in Supporting Information:
Brucella Immunogenic BP264 PDBid:id HVZ (Figure S1)
Haem-c-Cu nitrite reductase PDBid:id 4AX3 (Figure S2)
Nucleotide Complex of PyrR PDB id: 1NON (Figure S3)
Mammalian glutamate dehydrogenase, PDB id: 1NR7 (Figure S4)
Cu-adduct of human Ferritin, PDB id: 4DYX (Figure S5)
Biphenil-cleaving extradiol dioxygenase, PDB id: 1HAN (Figure S6)
Fungal prion, PDB id: 2RNM (Figure S7)
Tubulin-Kinesin Microtubule, PDB id: 3J2U (Figure S8)

## Discussion

In spite of not counting on a quantitative theory for the hydrophobic moment as simple and manageable as that for electric dipoles and thus being less intuitive, some important conclusions can be obtained when applied to the study of large structures. It is important to note is that the methods and results presented here are not intended to describe the peculiarities and details of the interactions of proteins when they associate, such as binding sites, kinetics, association rates, etc. Each association has its own idiosyncrasy and whatever these peculiarities may be they appear to obey the basic rules suggested in this paper.

**Figure 10:**
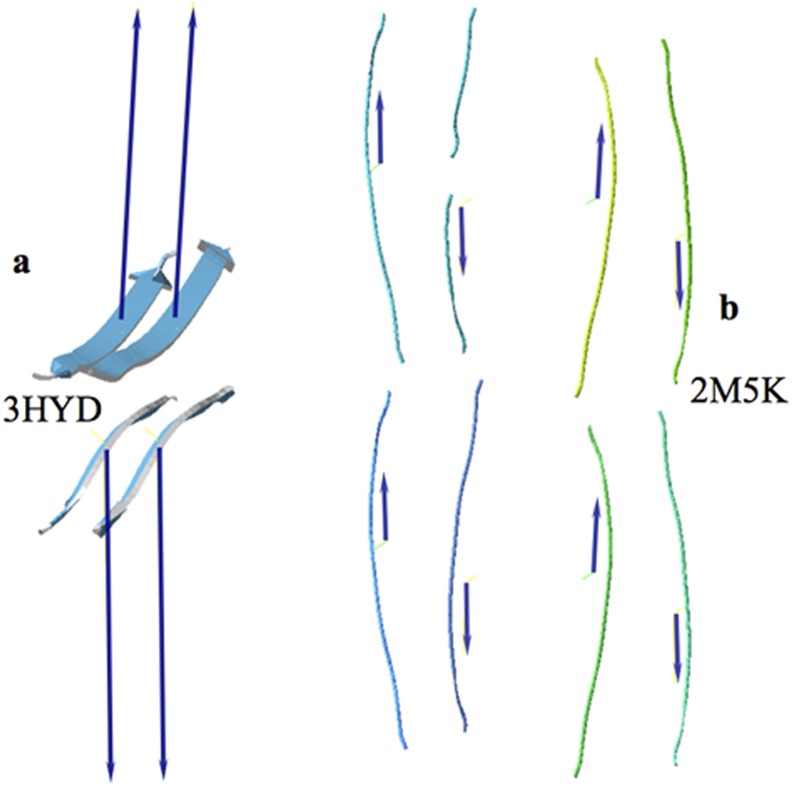
Assembly of amyloid peptides. a) A view of four parallel **NNQNTF** peptides [30] and their respective **H** vectors. These simple peptides associate side-by-side in arrays in opposite directions. b) Assembly of the doublet of cross-ß peptides **(YTIAALLSPYS)** [31], PDBid: 2M5K. Dark blue arrows represent the individual hydrophobic moments of each component of the doublet. Notice that the doublet is formed by two lobes of four peptides each. The components of the hydrophobic moments in the plane of the doublet cancel each other out leaving a negligible perpendicular component (not shown). Similar results are found in the triplet and quadruplet configurations. This association is somewhat more elaborate than that shown in a) since the distribution of **H** vectors combines opposite directions alternatively.

When hydrophobic moments are observed in a membrane from their constituent phospholipids, we expect them to form parallel alignments because of the alignment of the phospholipids and like hydrophobic moments of transmembrane proteins along with their phospholipid neighbors. When interpreting our results obtained in the aggregation of amyloid peptides, we see a striking analogy with the interaction of phospholipids within a membrane. The peptide amyloids beta loops described by Kim and Hecht [23] tend to associate and arrange in an analogous way as phospholipids do in a membrane: they tend to align their hydrophobic moments forming long arrays. Moreover, in the same way that it is not possible to have a single membrane monolayer in an aqueous solution, amyloid Aß_9–40_ peptide arrays and others cannot stay free in aqueous solutions and consequently they must join other arrays in an antiparallel orientation to each other, to form stable aggregates [23], [24], [30], [31] as seen in Figure 4. This simplest interpretation of the hydrophobic moment basically tells us that the hydrophobic centroids tend to attract each other while repelling hydrophilic centroids. It should be noted here that in these structures the electric dipole moments of the individual Aß_9–40_ peptide loops also tend to align with each other and in principle, as mentioned above, this means a repulsive interaction. At this point we cannot measure the electric and hydrophobic relative strengths, however we can conclude that the hydrophobic interactions of these species must overwhelm their opposing electric interactions. Similar conclusions can be drawn from amyloid peptides described by Eisenberg and Jucker [30] where no charged amino acids are involved. These authors studied the assembly of peptide **NNQNTF** (PDBid: 3HYD [30]) and others. Side-by-side association by aligning the hydrophobic moment vectors of two of the peptides can be appreciated in Figure 10a, where a disposition of the hydrophobic moments is totally reminiscent of that of phospholipids in a membrane.

Similar results can be observed for the association of peptide **YTIAALLSPYS** by Fitzpatrick et al. [31], when analyzing steric-zipper protofilaments. Starting with their protofilaments these authors were able to crystallize doublets, triplets and quadruplets arranged in planes in which the hydrophobic moments adopt a rather more complex pattern. Figure 10b shows one element of the stack in the doublet arrangement. In this case the individual hydrophobic moment vectors combine in alternating dispositions of parallel and antiparallel alignments, ready to accept a new doublet in the stack. Something similar can be said of their triplet and quadruplet associations.

**Figure 11:**
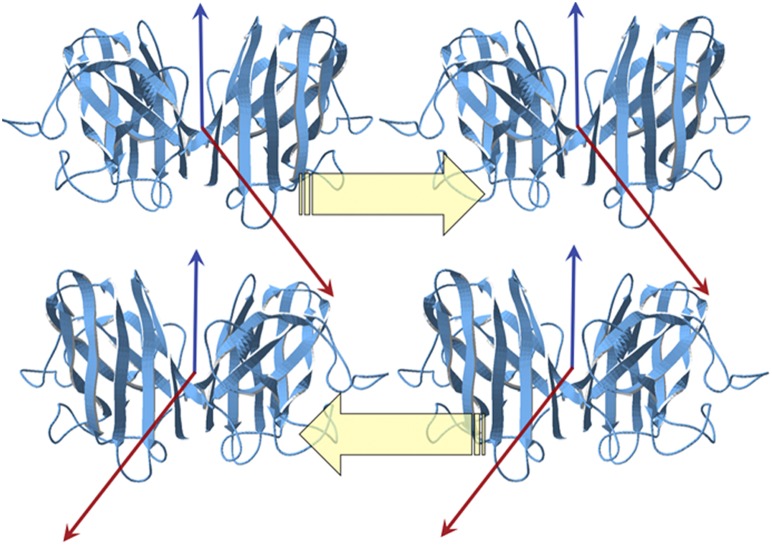
Postulated antiparallel arrangement of arrays of SOD1, as proposed in the text, in which two pairs of dimers form two different arrays. Red arrows represent electric dipole moments and blue arrows represent hydrophobic moments. Pale yellow arrows indicate the polarity of each linear association. This arrangement is both electrically and hydrophobically favorable for for a continuous growth: the individual D vectors lay at almost 90° of each other thus minimizing their interaction energy, whereas the H vectors align laterally. This disposition is in agreement with that given in [25].

SOD1 is a more complex protein and its self-assembly is driven by both hydrophobic and electric forces. The basic dimer is formed by hydrophobic effect, as seen in Results. The native protein (as a dimer) does not seem to oligomerize in significant amounts ‘in vivo’. However, either by suppression of its associated Ca^2+^ or Zn^2+^ ions or by mutations such as G93A, or by both, a significant horizontal component (perpendicular to the plane of the dimer) of the electric dipole moment comes out, suggesting a strong electric attraction towards other dimers. In Figure 11 we propose a way for these dimers to interact by alternating the polarity of the arrays.

This configuration is in agreement with that of “ß-cross” proposed by Seetharaman et al. [25].

Actin assembly is another example where electric interactions modulate the association since it is the external charges that provoke disruption of the arrangement of the electric dipole moments in the filament. The addition of a monomer to the actin filament is done in such a way that the **H** vector of the added monomer tends to align with that of the already formed filament (Figure 3), forming a narrow cone of hydrophobic moment vectors around the total **H** moment of the filament. The assembly is favored by the electric dipole moments disposition, since each **D** of the new monomer lies almost antiparallel to the preceding one. This is made possible because **H** and **D** of the total individual monomers are quasi-perpendicular to each other. Here, contrary to the amyloid association case, the electric interaction seems to play a cooperative role with the hydrophobic interaction. Changing the external electric conditions can disrupt this cooperativity and reverse the polymerization process. Under the adequate external conditions, actin filaments tend to associate side-by-side, induced by the tendency of their total **H** vectors to align side-by-side.

It is worth noting here that, independently of which particular series is chosen for analyzing the actin assembly in this study, the initial association of two monomers suggests a decrease of the modulus of the dipole moment, D. Since this dimer association corresponds to a particularly stable electric configuration, can it support the idea that the actin nucleation–elongation process starts with a nucleus of two dimers? Nevertheless, in spite of following both, amyloid association and actin polymerization, a nucleation-elongation mechanism of growth kinetics [32], [33], they show very different characteristics in their final structure due to the relative orientation of their **H** and **D** vectors.

A more complex polymerization process is that observed in the HIV-1 capsid when forming microtubules [26]. Although the mechanism is more elaborate, the result is similar to the above cases in terms of the net behavior of the hydrophobic moments. The strong **H** vectors associated to the basic hexamer units would provoke the formation of a layer of hexamers similar to phospholipids or amyloid layers, should this **H** vector be perfectly aligned with the hexamer axis. But slight folding differences among the components of the hexamer provoke their total **H** vectors to lay off the direction of the axis and then causing both a bend of the association and helicity in the growth. Figure 6 shows a spatial alignment of the components of the hexamer that serves as unit for growth of Human Immunodeficiency Virus-1 (HIV-1) capsid (PDBid: 3J4F). It is easy to observe different small folds in the six elements that may result in different **H** and **D** vectors.

**Figure 12:**
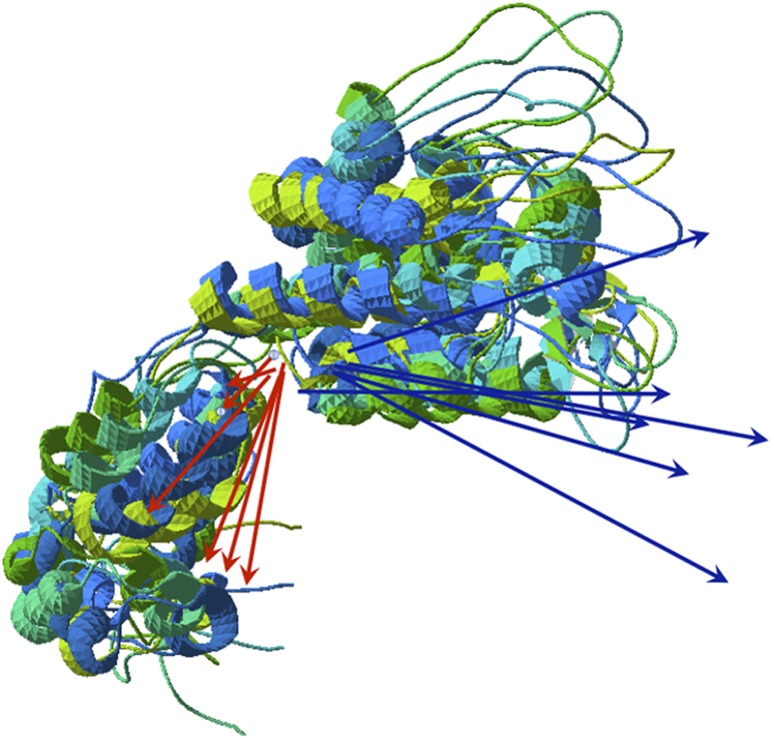
Structural heterogeneity of monomers in HIV capsid hexamers. Superposition of the six monomers that comprise the first hexamer of PDBid 3J4F. The variability in both moduli and direction of all six **D** (red) and **H** (blue) moments can be observed.

The result of this interaction is the precise combination of the relative orientation of the **H** and **D** vectors. As remarked in the Results section, seen from one side, the **D** vector on a hexamer seems almost perpendicular to the axis of the complex (less than 15°), whereas the **H** vector shows an appreciable deviation of about 20°. When two hexamers make the initial interaction to form the tubule, they tend to align their **H** vectors as expected. If only the hydrophobic interaction counted, a long straight line of hexamers would form. This oligomer would interact with another similar oligomer oriented in the opposite direction as seen in membranes or in amyloid formations. But the electric interaction also counts and seen from the axis of the tubule, the individual **D** vectors of the hexamers do not directly point to the axis but show a lateral component. These lateral components are crucial for the bending of the array of hexamers since the **D** vectors decrease their interaction energy as they adopt a more crossed over configuration. In other words, the bending of the array of hexamers is due to the tendency of the dipole moment vectors to close a circle, for which **D** → **0**. In addition to this, the variability of orientations of the **H** vectors (see Figure 12) does not allow the turn to close into a perfect circle, causing the appearance of a spiral thread and consequently further growth of the complex into a tubule.

Contrary to the case of open assembling systems, systems with limited number of monomers generally tend to decrease both their resultant **H** and **D** vectors. This fact provides a clue to deduce that in all spherically symmetric cases, H and D are actually zero or near zero. In spheroid systems like protein cages and virus capsid there seems to be a strong electric attraction among the monomers that is reinforced by the tendency to cancel the total hydrophobic moment of the system. In protein cages, the system has been designed in such a way that individual **H** vectors of the trimers are placed in a plane at about 120° of each other. Actually, many other cases (not reported here) render quite small total values of H and D as compared to those of their constituents which may be derived from small deviations from perfect symmetry and that may be used for other interactions. Such is the case of 1H2C, as shown in Results.

Other non-spherical systems (i.e. PDBid: 4HVZ) may also acquire a configuration in which H = 0 and D = 0, as long as a given structure can be complexed with another similar with opposing vectors.

A particularly interesting case is that of Ebola virus matrix protein VP40 (PDBid: 1H2C). The monomers in this octamer alternate their orientation and so do their respective **H** and **D** vectors. Such disposition of the individual electric dipole moments confers a very sound stability to this complex (**D** = **0**). The residual **H** vector in the axis suggests the addition of new rings in the direction of the axis in a similar manner as the case of amyloid peptides.

The data reported here suggests that it is the final tendency of the resultant hydrophobic moment that mainly determines the character of the association. For open associating systems, the addition of new elements on the complex increases the modulus of the resulting **H** vector. Conversely, in those non-growing cases with a limited number of monomers, the moduli of the resulting **H** vectors end up with values either zero or residual values as compared to those of their constituent monomers. Electric forces may play an important modulating role like in actin polymerization or may be the driving force for assembling as in the case of SOD1. The decrease of **D** upon assembly in most closed oligomerization cases certainly seems to be the rule, providing or reinforcing stability. In general, the data suggests that **H** may be cancelled in some particular direction, in which case no growth is expected, whereas it may keep increasing in the direction of growth. At this point it is clear that hydrophobic effects drive most protein association processes.

Table 1 describes the average dispersion of angles formed by the individual hydrophobic and electric dipole moments of compounds used in this study, around their total **H**_tot_ or **D**_tot_. Both open and closed systems have been listed. It can be observed that hydrophobic moments tend to align since the angles they form appear relatively small (35.8° ±5.8°). On the other hand, electric dipole moments adopt more disperse values (55.1° ±10.2°). D∧H angles may adopt all possible values (89.5° ±14.7°).

**Table 1:** Summary of angles of hydrophobic and electric dipole moments in compounds studied in this article.

Name	H_n_∧H_w_	D_n_∧D_w_	H∧D
1JS9	45.5	45.4	56.2
3J4F (hexamer)	15.7±1.8	34.4±2.4	133.5±2.5
3VCD (unit)	40.2±1.4	25.2±0.1	140.1±3.4
1H2C*	37.6	103.4	N/A
4HVZ*	50.9	35.9	32.4
4AX3	41.2	31.6	38.5
1NON	67.3	146.5	N/A
1NR7*	24.6	82.9	N/A
1HAN	76.5	89.8	N/A
2RNM	13.7±3.6	20.6±6.6	94.6±5.9
3J2U	10.4±0.6	64.9±2.4	56.2±6.4
1M8Q	28.9±3.4	82.5±9.6	88.4±1.4
2LMN (unit)	12.7±2.2	31.1±11.5	70.8±12.5

Note: H_n_∧H_w_ is the average angle (when applicable) formed by the hydrophobic moments of the whole compound (w) and those of the single elements (n). Same as for D_n_∧D_w_. (*) These compounds have some hydrophobic moments in opposite directions. Those larger than 90° have been subtracted from 180°, in order to show the relative small deviations from 180°. N/A: non applicable or not available.

An overall interpretation of the hydrophobic moment as defined in this article implies a two-step behavior in the assembly of proteins. First, in a given interaction between proteins, hydrophobic moments tend to align with a given efficiency depending on one hand on favorable or opposing electric interactions, and/or on more or less steric constraints on the other hand. Second, a tendency for the total hydrophobic moment of an assembled system to be counterbalanced with the total hydrophobic moment of another analogous assembled system, as is the situation in bilayer membranes, amyloid associations. When there is no such counterbalance, the system remains ready for more interaction and growth, as is the case of transmembrane proteins and actin polymers. It is common that two assembled systems can counterbalance each other in one or two directions but not in a third direction, provoking continuous growth in that particular direction, as in the case of amyloid growth.

## Conclusions

Given the membrane model for the alignment of **H** vectors, the tendency of hydrophobic vectors to align may be the main driving force that makes proteins associate to forms dimers, then trimers, etc. This is a consequence of the tendency of hydrophobic centroids to gather together. A general behavior pattern of **H** and **D** vectors emerges in this study, in which in open associations the intervening **H** vectors of the components tend to align, with a concomitant increase of **H**_total_ as new elements get incorporated into the assembly. Conversely, in closed systems **H**_total_ tend to decrease with respect to the individual hydrophobic vectors of the components, thus limiting the final number of components the system may acquire. In the end the total hydrophobic and dipole moments both tend to cancel out in situations of total equilibrium. The electric force may, in some cases, act as a modulating factor, facilitating the reversibility of some assembling processes in open systems, or providing stability in closed systems. These results do not seem to depend on the size of the proteins or complexes involved. This last point suggests that this analysis can be applied to any protein or assembly in the cell.

The model presented in this study does not intend to describe the fine individual anchoring details and mechanisms that govern all protein interactions. As previously mentioned, hydrogen bonds and other interactions are not being taken into account. This study doesn’t deal with the fact that proteins lay in specific ionic environments, which may have a direct influence in their characteristics for interaction. It is known that there are many requirements that must be met for two or more proteins to interact. What this work suggests is that whatever the specific mechanisms needed to assemble molecular machines that act in the cell may be, they comply with the specific electrical and hydrophobic principles presented in this article. The hydrophobic moments of proteins tend to align when they assemble within steric constrains. This work suggests that most associations are hydrophobically determined. Other assembly processes important for health and biotechnology are currently being studied using this methodology. The final goal is to predict the associative behavior of any protein or peptide. This will help design mutants to improve protein cages for biotechnology and help gain insight into the molecular basis of diseases caused by protein aggregation.

## Methods

### Oligomers and transmembrane proteins

A random set of 40 transmembrane proteins, both α– or ß– types, was used to compute their hydrophobic and electric dipole moments. Table S4 lists the PDB codes of these proteins chosen at random and their characteristics relevant to this study.

A number of protein associations such as actin polymers, amyloid aggregations or other associations, were obtained either from references in the literature or directly from the authors. The assemblies chosen in this study are the most representatives and conspicuous for the description of the model. Other systems are described in Supporting Information.

SwissPDViewer files were used for 3D visualization of all assemblies described in this article. Adding two alanines in the original PDB files in which Cα and N atoms depict the origin of the vector and the C and O depict the end of the vector graphically represent H and D vectors. PDB files showing the protein coordinates and their **H** and **D** vectors, are available from the authors on request.

### Pseudo Electric Dipole Moments

The classical definition of the electric dipole moment vector of a distribution of electric charges, is **D** = ∑q_j_.**r**_j_. **r_j_** are vector positions of charges q_j_, with respect to a chosen origin. Magnitudes in bold face represent vector quantities. Magnitudes in plain face design scalar quantities, such as the moduli of vectors.

These definitions depend on the origin of coordinates chosen unless the total charge (or total hydrophobicity) of a protein is zero. Given the fact that most proteins are not neutral (either electrically nor hydrophobically), we opted for other definitions, more practical for our purposes, by using the electric and hydrophobic centroids, defined in an analogous way as the centre of mass. In this way a pseudo-electric dipole moment vector **D**, is defined as **D** = (**c**_n_–**c**_p_).q^+^. In this expression **c**_n_ and **c**_p_ are the position vectors of the negative and positive centroids respectively, **c**_n_ = ∑q_j_.**r**_j_. and q^+^ is the total positive charge of the protein. In all figures depicting D vectors, the origin lies in positive centroid and the arrow points to the negative centroid. This definition coincides with the classical definition above, when the total charge of the protein is zero. The advantage of this definition is that it does not depend on the origin of coordinates and therefore it is an intrinsic parameter of the protein [16]. In what follows and for simplicity, this pseudo-electric dipole moment will be abbreviated as electric dipole moment or simply, dipole moment. Electric dipole moments are expressed in debyes.

### Hydrophobic Moments

Hydrophobic moments can be defined in a manner similar to the electric dipole moments, that is, **H** = ∑h_j_.**r**_j_, where h_j_ have been chosen to be the normalized values of the Eisenberg hydrophobicity scale for each amino acid [35]. This scale was chosen as the most widely used hydrophobicity scale. Some tests were carried out with other hydrophobicity scales and although different quantitative results were obtained, the qualitative tendencies of H vectors were the same. For the description of the hydrophobic character of the protein, we chose a definition of the hydrophobic moment that concerns only the “positive” hydrophobicity of the protein. The pseudo-hydrophobic moment of a protein is then defined as **H** = (**c** ^–^–**c** ^+^).h^+^. **c** ^+^ and **c** ^–^ are the positive (hydrophobic) and negative (hydrophilic) centroids of the protein and h^+^ is the total hydrophobicity of the protein, h^+^ = ∑h_j_. Again, bold face is used for vector magnitudes. Since hydrophobic moments are computed using the normalized Eisenberg hydrophobicity scale of values, hydrophobic moments are described in arbitrary units here called “rhu” (relative hydrophobic units) solely for the purpose of comparison. As in the case of dipole moments, the hydrophobic moments are depicted in this article with their origin in the hydrophobic centroids pointing towards the hydrophilic centroids. Our definition of a hydrophobic moment differs from that used by Eisenberg, which referrers to singular amino acids [34], [35], [36], intended to describe structural aspects within a protein. Our simpler definition is more suitable to describe the interaction of proteins. Another reason for using these definitions of pseudo moments (both electric and hydrophobic) is that they are more intuitive than the classical ones.

## Supporting Information

Figure S1 **Brucella Immunogenic BP26.** This is an example of an assembly in which both total **H** and **D** vectors are zero without it being a quasi-sphere. The channel-like membrane ensemble of proteins Brucella Immunogenic BP26 (PDBid: 4HVZ) described by Kim et al., cancels its total hydrophobic and dipolar moments out. The symetric disposition of the eight monomers in one half of the assembly (colored left half in a)) renders projections of the hydrophobic moments on the axis of the assembly as well on the plane perpendicular to the axis. In the latter case, all these components total zero, whereas components over the axis add to a vector over the axis of the ensemble. According to the membrane model, this would provide the octamer with a high propensity to stick to another octamer oriented in the opposite direction (grey in a)). Arrows show the individual hydrophobic moments of the octamer on the left (in color). **D** vectors which have not been depicted for clarity, follow a pattern similar to **H** vectors. Kim D, Park J, Kim SJ, Soh YM, Kim HM et al. (2013) Brucella Immunogenic BP26 Forms a Channel-like Structure. J Mol Biol 425: 1119–1126.
(TIF)

Figure S2 **Haem-c-Cu Nitrite Reductase.** This trimer (PDBid: 4AX3), described by Antonyuk et al. is interesting because it shows relatively large **H** (dark blue arrows) and **D** (red arrows) components in the direction of its axis (a), whereas the components of both **H** and **D** on the plane defined by the trimer (b) total zero, as in the former case. However, the complex is not known to assemble with other trimers, so the large resulting hydrophobic and dipole moments may be associated with other functions of the complex. Antonyuk S, Han C, Eady RR, Hasnain SS. (2013) Structures of protein–protein complexes involved in electron transfer. Nature 496: 123–127.
(TIF)

Figure S3 **Nucleotide Complex of PyrR.** This is the Pyr Attenuation Protein from Bacilus caldolyticus (PDBid: 1NON). This tetramer regulates the expression of genes and operons of pyrimidine nucleotide biosynthesis (pyr genes) in many bacteria. When active this protein acts as a dimer. In its unliganded state and the nucleotide-bound form, B. caldolyticus PyrR is a tetramer. In dimer form, there is a substantial decrease in the moduli of D (red arrows) and an increase in H (blue arrows) upon association. In tetramer form, both resultant H and D moduli decrease. The relative symetry of this complex, (like most similar structures) results in **H** and **D** vectors of moduli values of the same order of magnitude or smaller than the individual vectors of each monomer. Chandler P, Halbig KM, Miller JK, Fields CJ, Bonner HKS et al. (2005) Structure of the Nucleotide Complex of PyrR, the pyr Attenuation Protein from Bacillus caldolyticus. Suggests Dual Regulation by Pyrimidine and Purine Nucleotides. J Bacteriol 1773–1782.
(TIF)

Figure S4 **Mammalian Glutamate Dehydrogenase.** This complex (PDFid: 1NR7) is constituted by the assembly of six identical monomers and catalyzes the oxidative deamination of L-glutamate to 2-oxoglutarate. An interesting characteristic of this complex lies in the fact that the spatial distribution of its individual hydrophobic and dipole moments is not symmetrical but has a lopsided look as viewed from both the front plane (a) and from one side (b). The result is a net lateral component of the hydrophobic moment. a) dark blue arrows: hydrophobic moments of the monomers. b) purple arrows: electric dipole moments of the monomers. In both, black arrows are the resultant **H** vector; red arrow are the resultant **D** vector. According to Banerjee et al. these hexamers, when not interacting with their ligands, tend to aggregate in long polymers. c) representation of polymerisation mechanism of hexamers as **H** vectors have a parallel alignment, and **D** vectors tend to adopt a relative quasi perpendicular disposition with each other, as observed in [24]. Banerjee S, Schmidt T, Fang J, Stanley CA, Smith TJ. (2003) Structural Studies on ADP Activation of Mammalian Glutamate Dehydrogenase and the Evolution of Regulation. Biochemistry 42: 3446–3456.
(TIF)

Figure S5 **Cu-adduct of human Ferritin.** Another example of protein cages, is that obtained by the group of Tezcan, using reverse metal-template interface redesign (rMeTIR). These authors describe a copper-induced ferritin cage (PDBid: 4DYX) formed by 24 subunits by combining the adecuate mutations. In this case, each subunit is a quasi-paralel arrangement of alpha helices in which the hydrophobic moments are directed paralel to the helices, whereas the dipole moments form an angle of about 120° with **H**. This allows a tangencial disposition of the **H** vectors within the spheroid, with the **D** vectors directed towards the center. Note that each pair of helices (in color) have their hydrophobic centroids as close as possible to each other, given the steric limitations. Again, the resultant of **H** and **D** is zero. Huard DJE, Kane KM, Tezcan FA. (2013) Re-engineering protein interfaces yields copper-inducible ferritin cage assembly. Nat Chem Biol 9: 169–176.
(TIF)

Figure S6 **Biphenil-cleaving Extradiol Dioxygenase.** According to Han et al., this assembly (PDBid: 1HAN) is a dimer of tetramers disposed back-to-back, and its function is the biodegradation of aromatic pollutants. The structure looks like a hollow cylinder. In spite of its symmetric look. the dimers are not identical as far as their **H** and **D** vectors are concerned. As seen from the plane perpendicular to its axis (a), the components of both **H** and **D** cancel each other out leaving no resultant. However, along the axis of the cylinder (b), the components of the individual **H** vectors of one of the tetramers do show larger projection on the axis, yielding a net hydrophobic component. This case is an example of being D_tot_ = 0, but ∑H_i_ >> H_tot_ > H_i_. According to Han et al., this complex degradates contaminating biophenols. The fact that **H** is not zero may be the reason why these contaminants are attracted to the hollow of the cilynder to be dregraded there. Han S, Eltis LD, Timmis KN, Muchmore SW, Bolin JT. (1995) Crystal Structure of the Biphenyl-Cleaving Extradiol Dioxygenase from a PCB-Degrading Peudomonad. Science 270: 976–980.
(TIF)

Figure S7 **Fungal Prion.** Basic association of five peptides of fungal prions (PDBid: 2RNM) according to Smaoui et al. These authors propose different levels of association that resemble those described in [23], [24], [31]. a) Note the individual quasi-parallel green arrows that correspond to the individual hydrophobic moments of each basic peptide; blue vertical arrow is the **H** vector of the whole set. Red arrows correspond to the **D** vectors, essentially perpendicular to the H vectors. b) Same set vertically rotated 90°. According to Smaoui et al. these structures associate laterally forming a three element polygon. In this case both total **H** and **D** vectors would tend to anihilate in the most stable configuration. Smaoui M, Poitevin F, Delarue M, Koehl P, Orland H et al. (2013) Computational Assembly of Polymorphic Amyloid Fibrils Reveals Stable Aggregates. Biophys J 104: 683–693.
(TIF)

Figure S8 **Tubulin-Kinesin Microtubule.** Front (a) and lateral (b) views of the first turn of the tubulin-kinesin microtule (PDBid: 3J2U) according to Asenjo et al. Each turn is composed of 15 elements and each element is formed by two tubulin dimers linked through a kinesin molecule. Red arrows are the electric dipole moments of each element in the first turn and blue arrows are the individual **H** vectors. a) It is important to note the circular symmetry in the arrangement of the **D** vectors, making the component of **D**_tot_ in this plane almost zero. By contrast, individual **H** vectors seem to point in a single direction in this plane. Both **H**_tot_ and **D**_tot_ (large arrows in a) and b)) show components out of the plane. **D**_tot_ lies on the axis of the tubule and **H**_tot_ shows an off-axis component. It is likely that this assymetry may be the origin of the tendency to grow elliptically. The first 15 elements are depicted in green. The element colored in blue is the first of the next turn. For clarity, only a few individual **D** and **H** vectors are display in b). Asenjo A, Chaterjee C, Tan D, Depaoli V, Rice W et al. (2013) Structural model for tubulin recognition and deformation by kinesin-13 microtubule depolymerases. Cell Rep. 3: 759–768.
(TIF)

Table S1 **Vector characteristics of Actin dimers and oligomers.**
(DOC)

Table S2 **Vector characteristics of Aß_9–40_ amyloid associations and their Q15L mutation.**
(DOC)

Table S3 **Evolution of the polymerization of 3J4F (HIV capsid).**
(DOC)

Table S4 **Vector characteristics of transmembrane proteins used for survey.**
(DOC)

References S1 (DOCX)
