# Supplementary figures and images for: A Model of Protein Association Based on Their Hydrophobic and Electric Interactions
Source: PLoS One. 2014 Oct 17;9(10):e110352. doi: 10.1371/journal.pone.0110352 (PMC4201486; doi:10.1371/journal.pone.0110352)

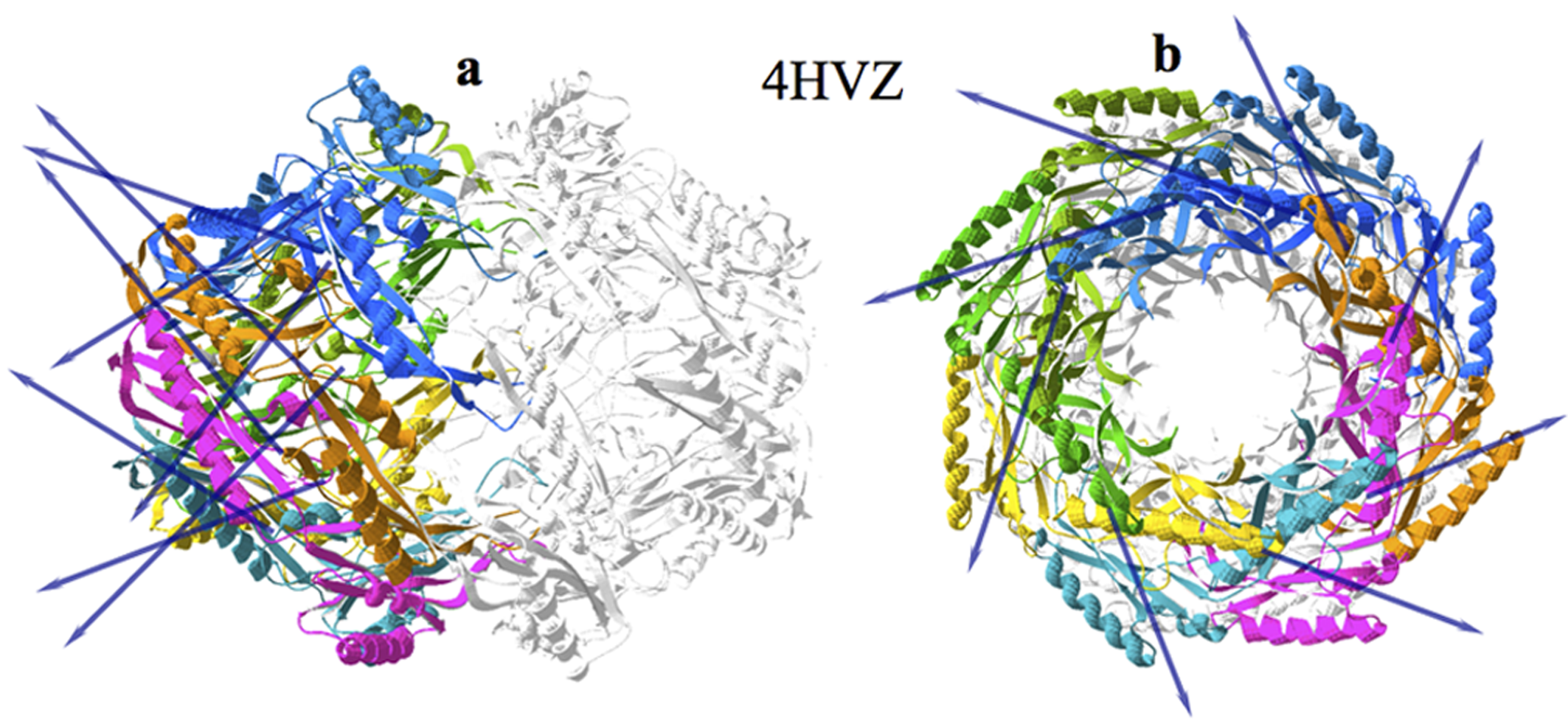

Supplement: Figure S1 — Brucella Immunogenic BP26. This is an example of an assembly in which both total H and D vectors are zero without it being a quasi-sphere. The channel-like membrane ensemble of proteins Brucella Immunogenic BP26 (PDBid: 4HVZ) described by Kim et al., cancels its total hydrophobic and dipolar moments out. The symetric disposition of the eight monomers in one half of the assembly (colored left half in a)) renders projections of the hydrophobic moments on the axis of the assembly as well on the plane perpendicular to the axis. In the latter case, all these components total zero, whereas components over the axis add to a vector over the axis of the ensemble. According to the membrane model, this would provide the octamer with a high propensity to stick to another octamer oriented in the opposite direction (grey in a)). Arrows show the individual hydrophobic moments of the octamer on the left (in color). D vectors which have not been depicted for clarity, follow a pattern similar to H vectors. Kim D, Park J, Kim SJ, Soh YM, Kim HM et al. (2013) Brucella Immunogenic BP26 Forms a Channel-like Structure. J Mol Biol 425: 1119–1126. (TIF) [file pone.0110352.s001.tif]

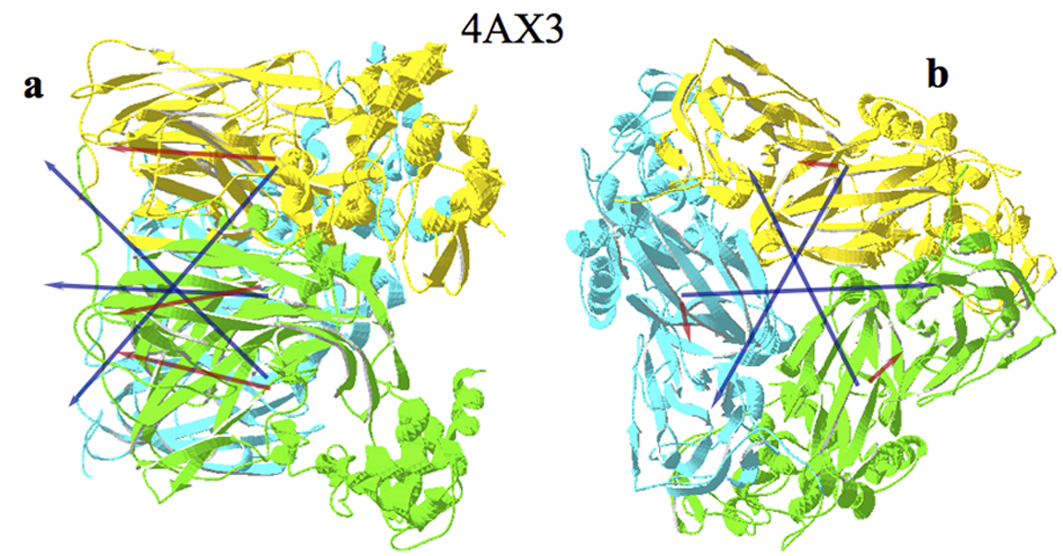

Supplement: Figure S2 — Haem-c-Cu Nitrite Reductase. This trimer (PDBid: 4AX3), described by Antonyuk et al. is interesting because it shows relatively large H (dark blue arrows) and D (red arrows) components in the direction of its axis (a), whereas the components of both H and D on the plane defined by the trimer (b) total zero, as in the former case. However, the complex is not known to assemble with other trimers, so the large resulting hydrophobic and dipole moments may be associated with other functions of the complex. Antonyuk S, Han C, Eady RR, Hasnain SS. (2013) Structures of protein–protein complexes involved in electron transfer. Nature 496: 123–127. (TIF) [file pone.0110352.s002.tif]

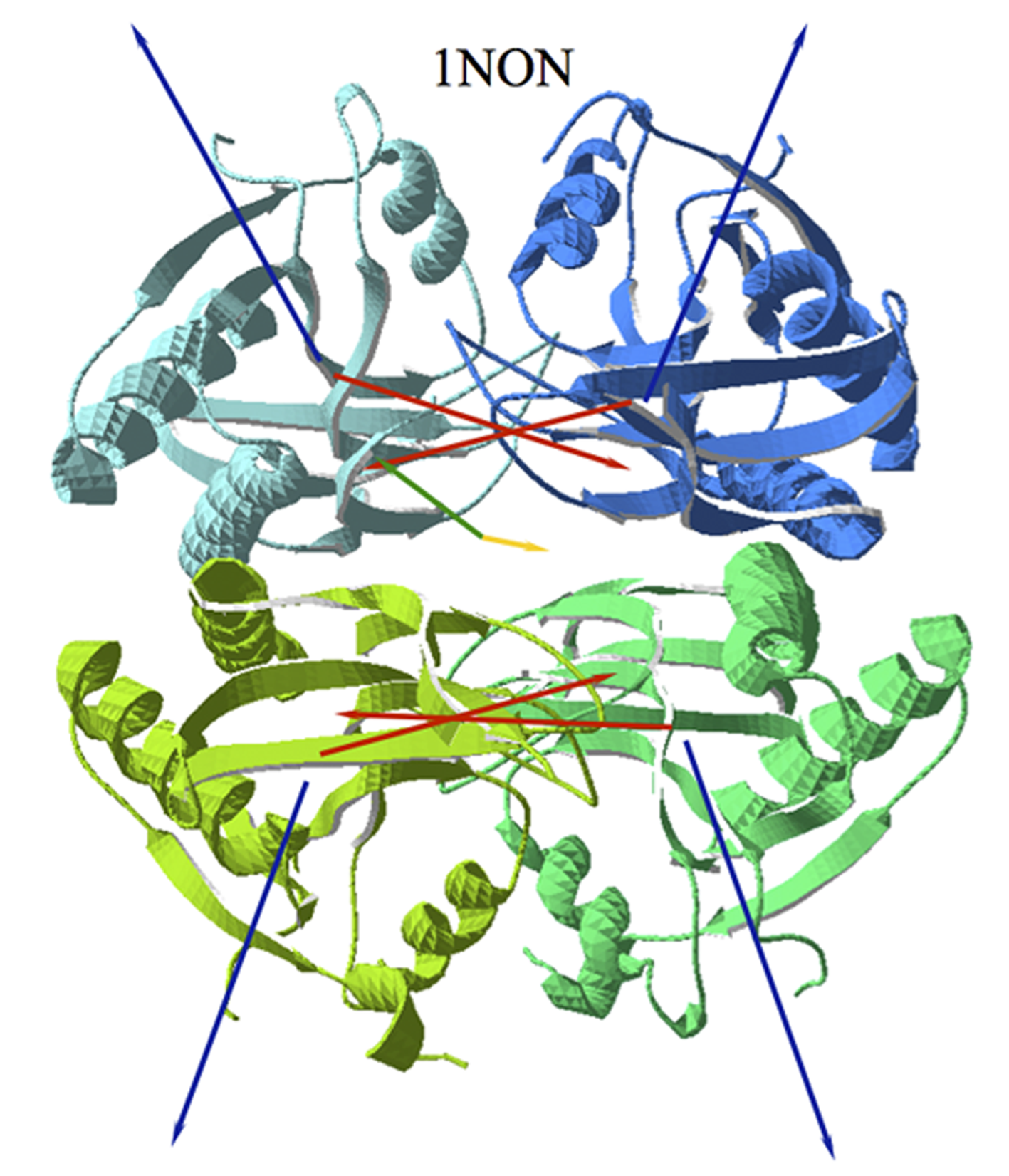

Supplement: Figure S3 — Nucleotide Complex of PyrR. This is the Pyr Attenuation Protein from Bacilus caldolyticus (PDBid: 1NON). This tetramer regulates the expression of genes and operons of pyrimidine nucleotide biosynthesis (pyr genes) in many bacteria. When active this protein acts as a dimer. In its unliganded state and the nucleotide-bound form, B. caldolyticus PyrR is a tetramer. In dimer form, there is a substantial decrease in the moduli of D (red arrows) and an increase in H (blue arrows) upon association. In tetramer form, both resultant H and D moduli decrease. The relative symetry of this complex, (like most similar structures) results in H and D vectors of moduli values of the same order of magnitude or smaller than the individual vectors of each monomer. Chandler P, Halbig KM, Miller JK, Fields CJ, Bonner HKS et al. (2005) Structure of the Nucleotide Complex of PyrR, the pyr Attenuation Protein from Bacillus caldolyticus. Suggests Dual Regulation by Pyrimidine and Purine Nucleotides. J Bacteriol 1773–1782. (TIF) [file pone.0110352.s003.tif]

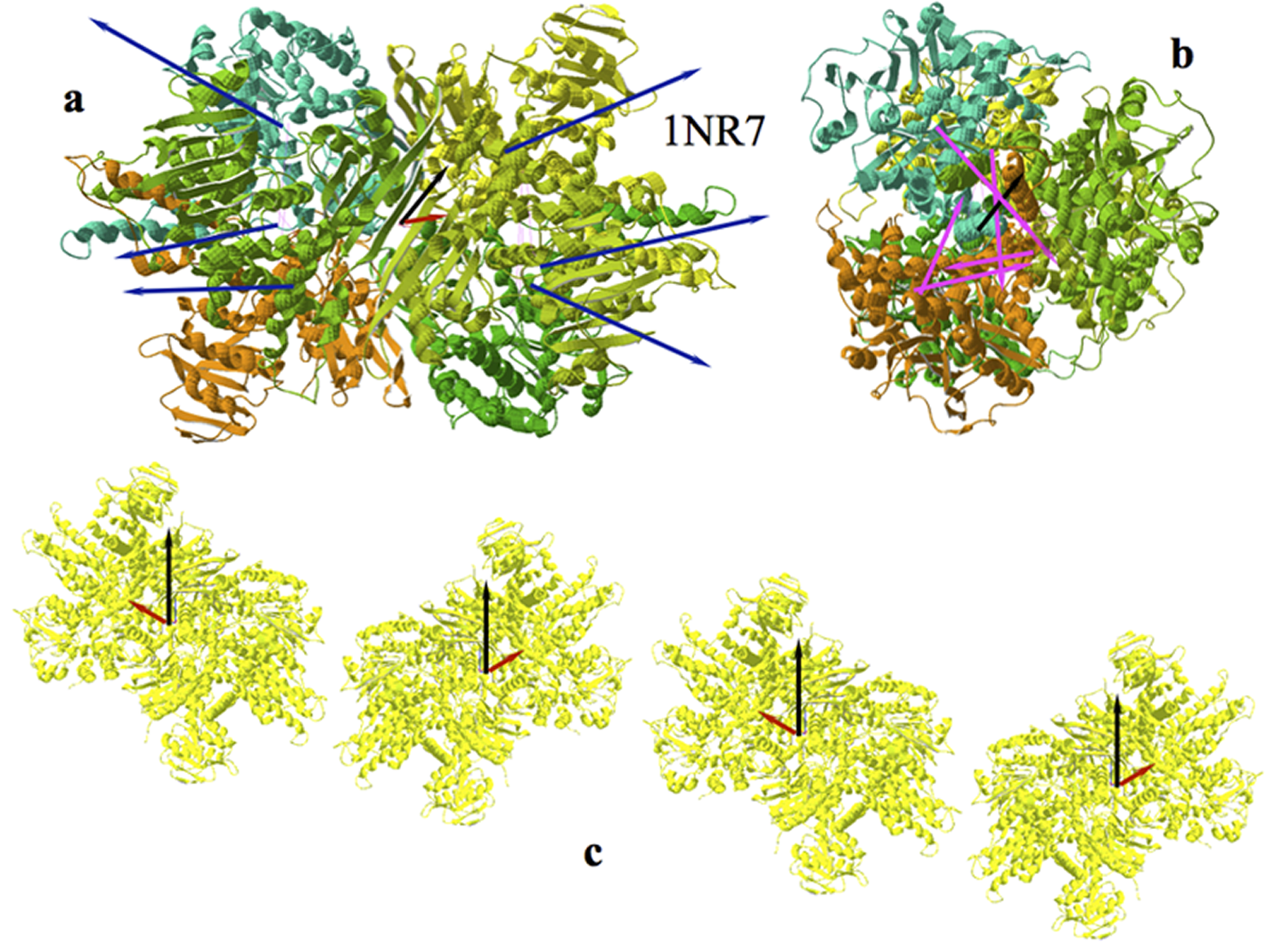

Supplement: Figure S4 — Mammalian Glutamate Dehydrogenase. This complex (PDFid: 1NR7) is constituted by the assembly of six identical monomers and catalyzes the oxidative deamination of L-glutamate to 2-oxoglutarate. An interesting characteristic of this complex lies in the fact that the spatial distribution of its individual hydrophobic and dipole moments is not symmetrical but has a lopsided look as viewed from both the front plane (a) and from one side (b). The result is a net lateral component of the hydrophobic moment. a) dark blue arrows: hydrophobic moments of the monomers. b) purple arrows: electric dipole moments of the monomers. In both, black arrows are the resultant H vector; red arrow are the resultant D vector. According to Banerjee et al. these hexamers, when not interacting with their ligands, tend to aggregate in long polymers. c) representation of polymerisation mechanism of hexamers as H vectors have a parallel alignment, and D vectors tend to adopt a relative quasi perpendicular disposition with each other, as observed in [24]. Banerjee S, Schmidt T, Fang J, Stanley CA, Smith TJ. (2003) Structural Studies on ADP Activation of Mammalian Glutamate Dehydrogenase and the Evolution of Regulation. Biochemistry 42: 3446–3456. (TIF) [file pone.0110352.s004.tif]

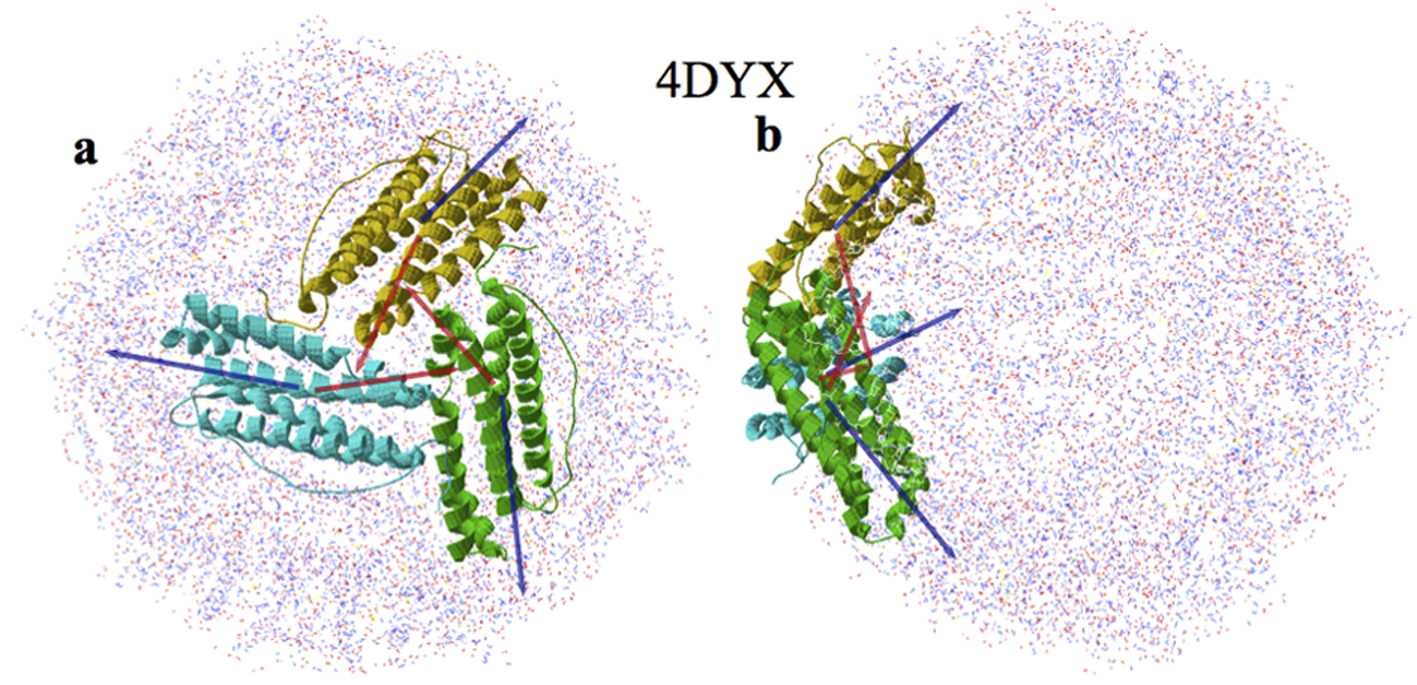

Supplement: Figure S5 — Cu-adduct of human Ferritin. Another example of protein cages, is that obtained by the group of Tezcan, using reverse metal-template interface redesign (rMeTIR). These authors describe a copper-induced ferritin cage (PDBid: 4DYX) formed by 24 subunits by combining the adecuate mutations. In this case, each subunit is a quasi-paralel arrangement of alpha helices in which the hydrophobic moments are directed paralel to the helices, whereas the dipole moments form an angle of about 120° with H. This allows a tangencial disposition of the H vectors within the spheroid, with the D vectors directed towards the center. Note that each pair of helices (in color) have their hydrophobic centroids as close as possible to each other, given the steric limitations. Again, the resultant of H and D is zero. Huard DJE, Kane KM, Tezcan FA. (2013) Re-engineering protein interfaces yields copper-inducible ferritin cage assembly. Nat Chem Biol 9: 169–176. (TIF) [file pone.0110352.s005.tif]

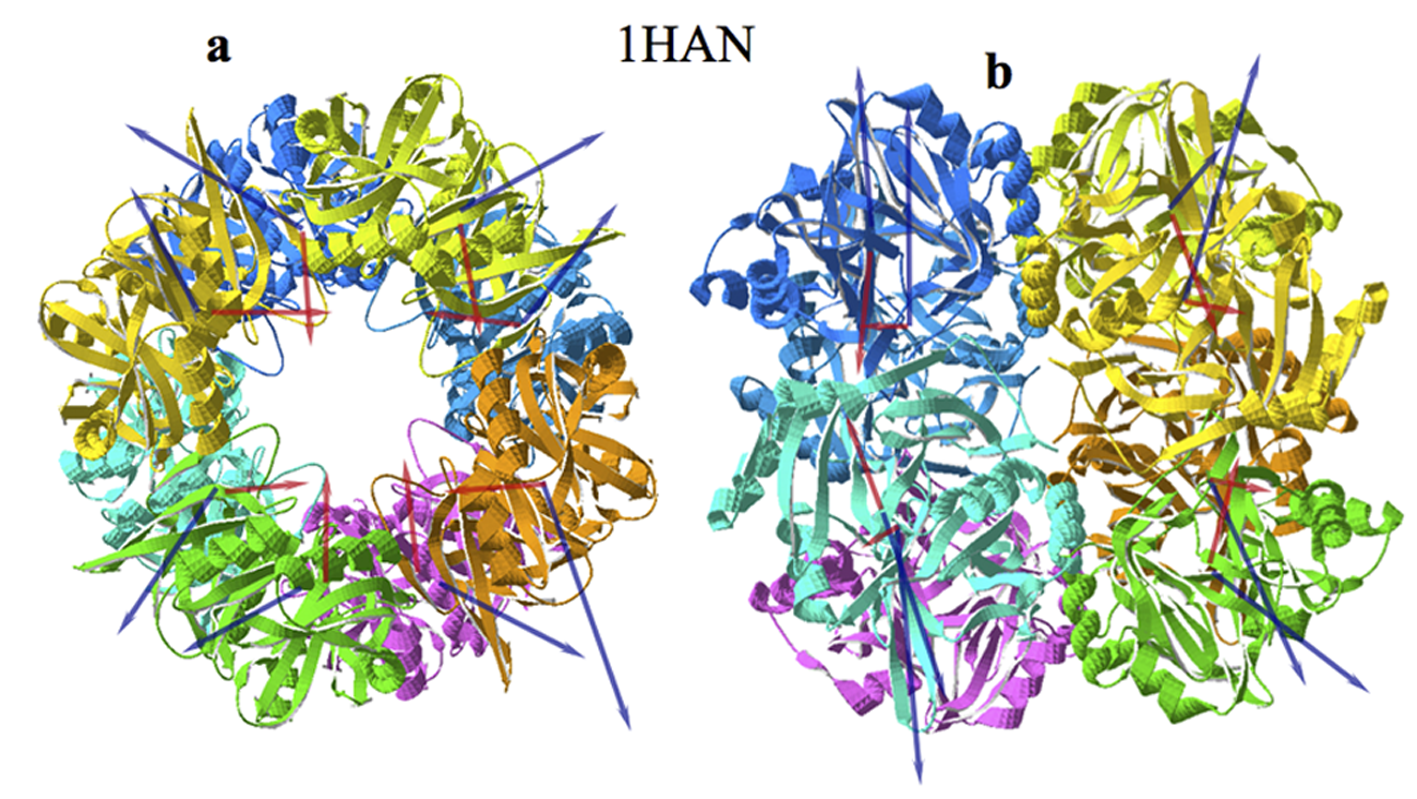

Supplement: Figure S6 — Biphenil-cleaving Extradiol Dioxygenase. According to Han et al., this assembly (PDBid: 1HAN) is a dimer of tetramers disposed back-to-back, and its function is the biodegradation of aromatic pollutants. The structure looks like a hollow cylinder. In spite of its symmetric look. the dimers are not identical as far as their H and D vectors are concerned. As seen from the plane perpendicular to its axis (a), the components of both H and D cancel each other out leaving no resultant. However, along the axis of the cylinder (b), the components of the individual H vectors of one of the tetramers do show larger projection on the axis, yielding a net hydrophobic component. This case is an example of being Dtot = 0, but ∑Hi >> Htot > Hi. According to Han et al., this complex degradates contaminating biophenols. The fact that H is not zero may be the reason why these contaminants are attracted to the hollow of the cilynder to be dregraded there. Han S, Eltis LD, Timmis KN, Muchmore SW, Bolin JT. (1995) Crystal Structure of the Biphenyl-Cleaving Extradiol Dioxygenase from a PCB-Degrading Peudomonad. Science 270: 976–980. (TIF) [file pone.0110352.s006.tif]

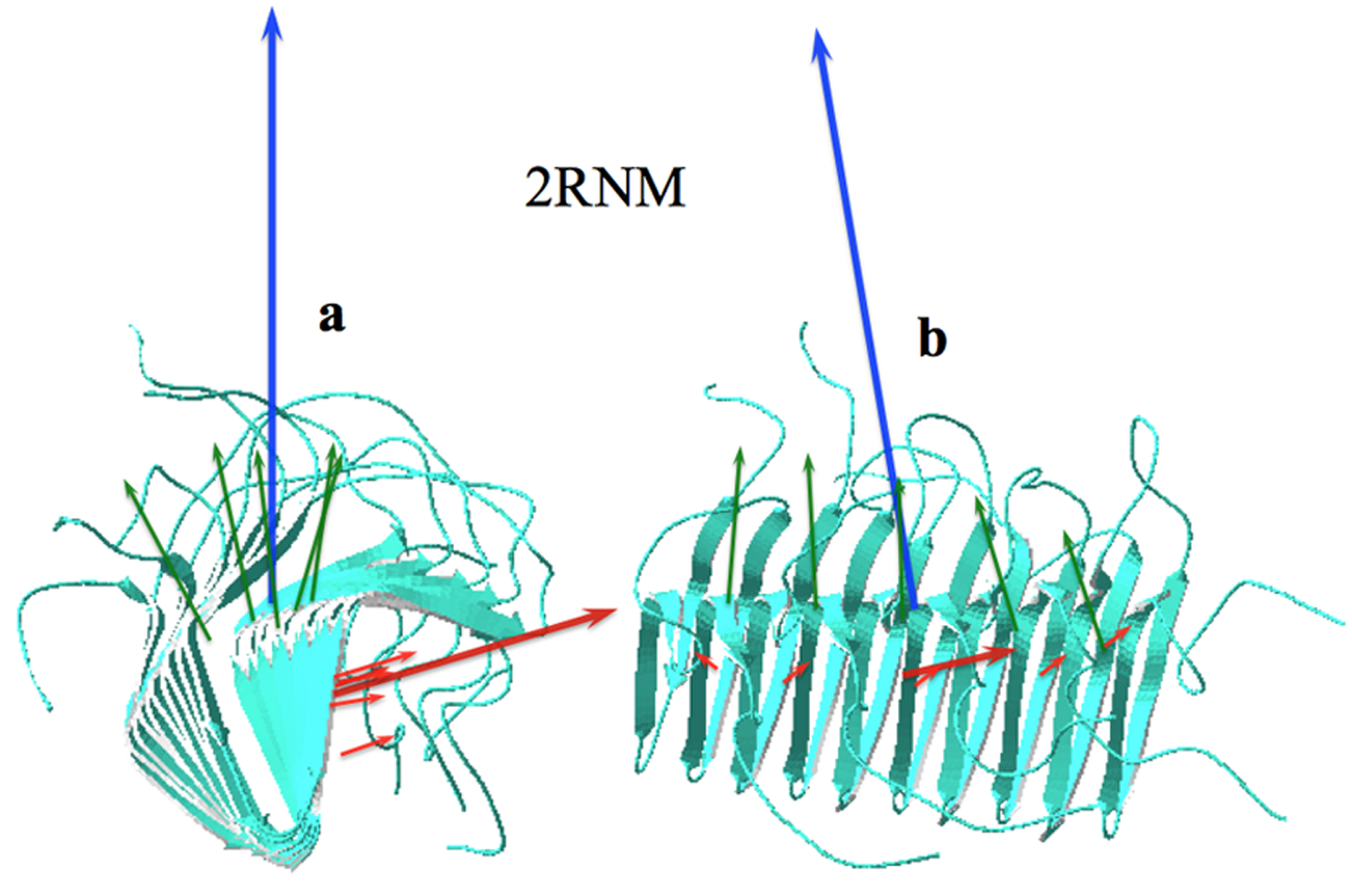

Supplement: Figure S7 — Fungal Prion. Basic association of five peptides of fungal prions (PDBid: 2RNM) according to Smaoui et al. These authors propose different levels of association that resemble those described in [23], [24], [31]. a) Note the individual quasi-parallel green arrows that correspond to the individual hydrophobic moments of each basic peptide; blue vertical arrow is the H vector of the whole set. Red arrows correspond to the D vectors, essentially perpendicular to the H vectors. b) Same set vertically rotated 90°. According to Smaoui et al. these structures associate laterally forming a three element polygon. In this case both total H and D vectors would tend to anihilate in the most stable configuration. Smaoui M, Poitevin F, Delarue M, Koehl P, Orland H et al. (2013) Computational Assembly of Polymorphic Amyloid Fibrils Reveals Stable Aggregates. Biophys J 104: 683–693. (TIF) [file pone.0110352.s007.tif]

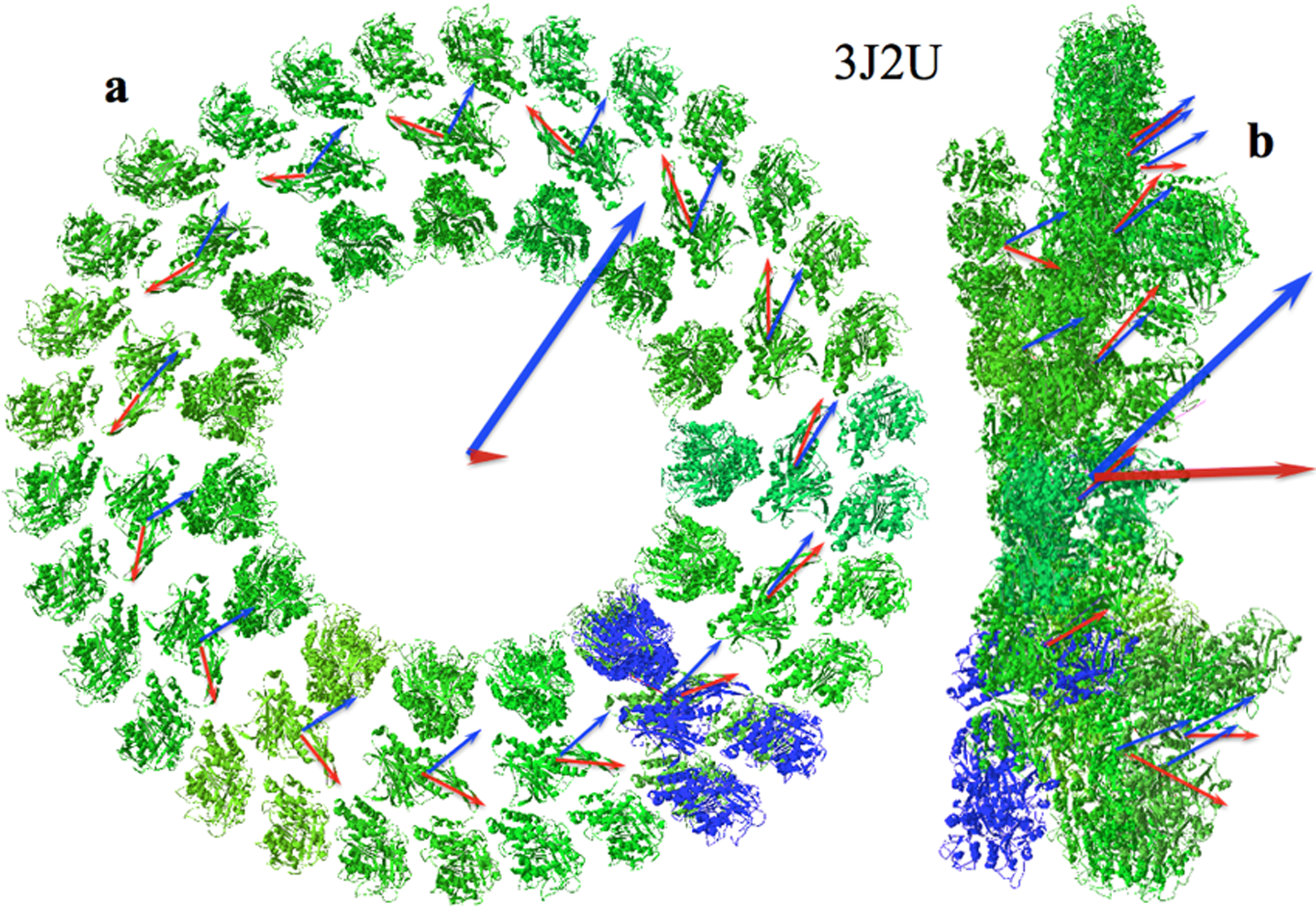

Supplement: Figure S8 — Tubulin-Kinesin Microtubule. Front (a) and lateral (b) views of the first turn of the tubulin-kinesin microtule (PDBid: 3J2U) according to Asenjo et al. Each turn is composed of 15 elements and each element is formed by two tubulin dimers linked through a kinesin molecule. Red arrows are the electric dipole moments of each element in the first turn and blue arrows are the individual H vectors. a) It is important to note the circular symmetry in the arrangement of the D vectors, making the component of D tot in this plane almost zero. By contrast, individual H vectors seem to point in a single direction in this plane. Both H tot and D tot (large arrows in a) and b)) show components out of the plane. D tot lies on the axis of the tubule and H tot shows an off-axis component. It is likely that this assymetry may be the origin of the tendency to grow elliptically. The first 15 elements are depicted in green. The element colored in blue is the first of the next turn. For clarity, only a few individual D and H vectors are display in b). Asenjo A, Chaterjee C, Tan D, Depaoli V, Rice W et al. (2013) Structural model for tubulin recognition and deformation by kinesin-13 microtubule depolymerases. Cell Rep. 3: 759–768. (TIF) [file pone.0110352.s008.tif]
