# Supplementary material for: A Model of Protein Association Based on Their Hydrophobic and Electric Interactions
Source: PLoS One. 2014 Oct 17;9(10):e110352. doi: 10.1371/journal.pone.0110352 (PMC4201486; doi:10.1371/journal.pone.0110352)
Supplement: Table S1 — Vector characteristics of Actin dimers and oligomers. (DOC) [file pone.0110352.s009.doc]

Table S1. Vector characteristics of Actin dimers and oligomers

| name | sequence | D | H | H^D | H^H | D^D |
| --- | --- | --- | --- | --- | --- | --- |
| 1ATN | 1–372 | 41.3 | 118.7 | 70.5 |  |  |
| 1Y64 | 4–372 | 37.3 | 207.9 | 35.8 |  |  |
| 2ZWH | 1–375 | 21.0 | 139.5 | 76.9 |  |  |
| 2GWJ | 5–375 | 14.5 | 159.9 | 36.5 |  |  |
| 3UB5 | 6–375 | 12.5 | 155.9 | 141.8 |  |  |
| 3HBT | 5–375 | 16.4 | 158.2 | 34.9 |  |  |
| 1J6Z | 4–372 | 16.1 | 119.0 | 112.3 |  |  |
| average |  | 22.7±4.4 | 151.3±11.5 | 72.7±15.8 |  |  |
|  |  |  |  |  |  |  |
| 1M8Q |  |  |  |  |  |  |
| Actin0 | 1–372 | 24.38 | 140.7 | 92.9 |  |  |
| Actin1 | 1–372 | 24.39 | 144.8 | 92.5 |  |  |
| Actin2 | 1–372 | 24.27 | 149.1 | 94.4 |  |  |
| Actin3 | 1–372 | 24.32 | 154.9 | 93.1 |  |  |
| Actin4 | 1–372 | 24.2 | 158.3 | 87.2 |  |  |
| Actin5 | 1–372 | 24.25 | 164.8 | 93.8 |  |  |
|  |  |  |  |  |  |  |
| Actin7 | 1–372 | 24.75 | 105.8 | 89.4 |  |  |
| Actin8 | 1–372 | 24.66 | 107.3 | 88.8 |  |  |
| Actin9 | 1–372 | 24.65 | 114.1 | 88.7 |  |  |
|  |  |  |  |  |  |  |
| ActinV | 1–372 | 24.6 | 116.3 | 90.0 |  |  |
| ActinW | 1–372 | 24.5 | 122.5 | 90.7 |  |  |
| ActinX | 1–372 | 24.5 | 125.5 | 91.1 |  |  |
| ActinY | 1–372 | 24.5 | 131.3 | 92.1 |  |  |
| ActinZ | 1–372 | 24.4 | 134.8 | 92.1 |  |  |
| 3J0S |  |  |  |  |  |  |
| Actin a | 2–375 | 23.7 | 174.3 | 73.7 |  |  |
| Actin b | 2–375 | 23.7 | 169.7 | 79.1 |  |  |
| Actin c | 2–375 | 23.7 | 165.3 | 79.3 |  |  |
| Actin d | 2–375 | 23.6 | 160.8 | 98.4 |  |  |
| Actin e | 2–375 | 23.6 | 156.4 | 79.8 |  |  |
| Actin f | 2–375 | 23.6 | 151.9 | 80.1 |  |  |
| 0+1 |  | 22.8 | 259.4 |  | H_0_^H_1_ = 49.4 | D_0_^D_1_ = 118.0 |
| 0+1+2 |  | 31.1 | 394.2 |  | H_01_^H_2_ = 31.6 | D_01_^D_2_ = 97.3 |
| 0+1+2+3 |  | 39.4 | 535.5 |  | H_012_^H_3_ = 28.5 | D_012_^D_3_ = 90.2 |
| 0+1+2+3+4 |  | 44.1 | 678.8 |  | H_0123_^H_4_ = 28.6 | D_0123_^D_4_ = 95.7 |
| 0+1+2+3+4+5 |  | 55.7 | 832.7 |  | H_01234_^H_5_ = 23.2 | D_01234_^D_5_ = 74.5 |
| 7+8 |  | 19.7 | 172.2 |  | H_7_^H_8_ = 72.2 | D_7_^D_8_ = 132.9 |
| 7+8+9 |  | 35.7 | 273.4 |  | H_78_^H_9_ = 35.2 | D_78_^D_9_ = 73.2 |
| V+W |  | 19.0 | 203.3 |  | H_V_^H_W_ = 63.3 | D_V_^D_W_ = 134.4 |
| V+W+X |  | 35.5 | 317.5 |  | H_VW_^H_X_ = 31.1 | D_VW_/D_X_ = 71.6 |
| V+W+X+Y |  | 37.4 | 427.4 |  | H_VWX_^H_Y_ = 28.0 | D_VWX_/D_Y_ = 66.1 |
| V+W+X+Y+Z |  | 48.8 | 513.2 |  | H_VWXY_^H_Z_ = 28.7 | D_VWXY_^D_Z_ = 71.5 |
| a+b |  | 11.77 | 304.8 |  | H_a_^H_b_ = 55.2 | D_a_^D_b_ = 151.2 |
| a+b+c |  | 25.13 | 453.8 |  | H_ab_^H_c_ = 31.7 | D_ab_^D_c_ = 96.9 |
| a+b+c+d |  | 22.86 | 588.9 |  | H_abc_^H_d_ = 37.5 | D_abc_^D_d_ = 124.2 |
| a+b+c+d+e |  | 28.12 | 723.1 |  | H_abcd_^H_e_ = 34.5 | D_abcd_^D_e_ = 105.6 |
| a+b+c+d+e+f |  | 32.87 | 852.1 |  | H_abcde_^H_f_ = 35.0 | D_abcde_^D_f_ = 101.6 |

Note: Numerals (0 to 5 and 7 to 9) and letters (V to Z) serve to identify the monomers in the complexes given in [21]. Letters a to f are identifiers given in [22]
