# Supplementary material for: A Model of Protein Association Based on Their Hydrophobic and Electric Interactions
Source: PLoS One. 2014 Oct 17;9(10):e110352. doi: 10.1371/journal.pone.0110352 (PMC4201486; doi:10.1371/journal.pone.0110352)
Supplement: Table S2 — Vector characteristics of Aß9–40 amyloid associations and their Q15L mutation. (DOC) [file pone.0110352.s010.doc]

Table S2. Vector characteristics of Aß_9–40_ amyloid associations and their Q15L mutation

| NAME | UNIT | D | H | H^D | H^H | D^D |
| --- | --- | --- | --- | --- | --- | --- |
| 2LMN | T | 11.12 | 215.6 | 9.4 | H_1_^H_2_ = 107.9 | D_1_^D_2_ = 144.5 |
| 2LMN1 | 1 | 18.29 | 186.5 | 81.4 |  |  |
|  | a | 11.45 | 39.5 | 59.4 |  |  |
|  | b | 0.64 | 34.5 | 17.8 |  |  |
|  | c | 2.95 | 34.2 | 91.5 |  |  |
|  | d | 5.36 | 37.6 | 82.4 |  |  |
|  | e | 4.54 | 25.9 | 68.1 |  |  |
|  | f | 5.02 | 19.9 | 105.5 |  |  |
|  |  |  |  | 70.8±12.5 |  |  |
| 2LMN2 | 2 | 18.25 | 179.8 | 93.8 |  |  |
|  | g | 1.44 | 38.2 | 152.7 |  |  |
|  | h | 1.67 | 28.5 | 109.5 |  |  |
|  | i | 3.47 | 39.9 | 99.5 |  |  |
|  | j | 3.79 | 29.4 | 83.3 |  |  |
|  | k | 3.96 | 27.2 | 80.6 |  |  |
|  | l | 5.37 | 25.3 | 88.6 |  |  |
|  |  |  |  | 102.4±11.0 |  |  |
|  |  |  |  |  |  |  |
| Q15L | T | 11.13 | 163.4 | 12.3 | H_1_^H_2_ = 128.1 | D_1_^D_2_ = 144.5 |
| Q15L1 | 1 | 18.30 | 194.2 | 69.8 |  |  |
|  | a | 1.45 | 40.6 | 54.2 |  |  |
|  | b | 0.65 | 38.1 | 22.5 |  |  |
|  | c | 2.95 | 35.4 | 80.9 |  |  |
|  | d | 5.37 | 36.4 | 72.0 |  |  |
|  | e | 4.55 | 28.5 | 62.1 |  |  |
|  | f | 5.03 | 20.4 | 81.1 |  |  |
|  |  |  |  | 62.1±9.0 |  |  |
| Q15L2 | 2 | 18.25 | 177.9 | 80.4 |  |  |
|  | g | 1.43 | 38.3 | 134.8 |  |  |
|  | h | 1.67 | 28.5 | 109.5 |  |  |
|  | i | 3.47 | 36.1 | 89.1 |  |  |
|  | j | 3.78 | 32.3 | 73.2 |  |  |
|  | k | 3.96 | 28.8 | 63.5 |  |  |
|  | l | 5.37 | 23.9 | 64.1 |  |  |
|  |  |  |  | 89.0±11.6 |  |  |

Note: 2LMN1 and 2LMN2 are the two sets of amyloid filaments that constitute PDBid: 2LMN. Q15L1 and Q15L2 are the corresponding sets with the Q15L mutations [23,24].
