# Supplementary material for: A Model of Protein Association Based on Their Hydrophobic and Electric Interactions
Source: PLoS One. 2014 Oct 17;9(10):e110352. doi: 10.1371/journal.pone.0110352 (PMC4201486; doi:10.1371/journal.pone.0110352)
Supplement: Table S3 — Evolution of the polymerization of 3J4F (HIV capsid). (DOC) [file pone.0110352.s011.doc]

Table S3. Evolution of the polymerization of 3J4F ( HIV capsid)

| Hexamers/ turn | D | H | H_n_^H_w_ | D_n_^D_w_ |  |
| --- | --- | --- | --- | --- | --- |
| a | 137 | 534 |  |  |  |
| ab | 264 | 1031 | 28.7 | 31.1 |  |
| abc | 373 | 1460 | 43.1 | 46.7 |  |
| abcd | 454 | 1796 | 57.4 | 62.2 |  |
| abcde | 502 | 2022 | 71.4 | 77.8 |  |
| abcdef | 513 | 2131 | 84.8 | 93.3 |  |
| abcdefg | 486 | 2130 | 97.0 | 108.9 |  |
| abcdefgh | 423 | 2041 | 106.9 | 124.4 |  |
| abcdefghi | 330 | 1904 | 112.7 | 139.8 |  |
| abcdefghij | 214 | 1779 | 111.8 | 154.8 |  |
| abcdefghijk | 86 | 1736 | 103.1 | 166.5 |  |
| abcdefghijkl | 72 | 1818 | 88.6 | 152.9 |  |
| Hexamer | D | H | H^H_a_ | D^D_a_ | H^D |
| 3J4Fa | 137.7 | 534.3 |  |  | 157.1 |
| a1 | 29.1 | 92.9 | 166.4 | 169.1 | 144.5 |
| a2 | 28.9 | 105.6 | 159.3 | 152.8 | 117.4 |
| a3 | 31.5 | 88.3 | 159.1 | 131.2 | 118.5 |
| a4 | 26.3 | 101.9 | 146.1 | 129.7 | 131.4 |
| a5 | 24.0 | 85.5 | 153.0 | 115.1 | 134.1 |
| a6 | 27.6 | 78.3 | 157.3 | 145.7 | 151.2 |
| average (6) | 29.9±1.1 | 92.1±4.2 | 156.9±2.8 | 140.6±7.8 | 132.8±5.5 |

Note: H_n_^H_w_ is the angle formed by the hydrophobic moment of the already formed compound (w) and that of the new added element (n). Same as for D_n_^D_w_. D is in debyes, H is in rhu (see text).

Values corresponding to the six components of the first hexamer (a) that constitutes the HIV capsid of PDBid: 3J4F [26].
