# Supplementary material for: A Model of Protein Association Based on Their Hydrophobic and Electric Interactions
Source: PLoS One. 2014 Oct 17;9(10):e110352. doi: 10.1371/journal.pone.0110352 (PMC4201486; doi:10.1371/journal.pone.0110352)
Supplement: Table S4 — Vector characteristics of transmembrane proteins used for survey. (DOC) [file pone.0110352.s012.doc]

Table S4. Vector characteristics of transmembrane proteins used for survey

| Name | D | H | H^D | Type |
| --- | --- | --- | --- | --- |
| 1A0S | 70.6 | 1053.6 | 2.8 | β |
| 1BH3 | 96.0 | 274.5 | 5.4 | β |
| 1BT9 | 21.0 | 680.6 | 176.8 | β |
| 1BXW | 21.5 | 126.6 | 164 | β |
| 1BY5 | 36.2 | 285.0 | 125.6 | β |
| 1GZM | 52.4 | 94.2 | 146.8 | α |
| 1J7F | 16.6 | 6.39 | 139.9 | α |
| 1KMO | 60.1 | 556.0 | 150.1 | β |
| 1ORM | 14.8 | 143.2 | 153.5 | coil |
| 1PY6 | 9.4 | 96.0 | 114.5 | α |
| 1RWT | 61.0 | 335.3 | 1.03 | α |
| 1S5L | 817.4 | 7490.1 | 1.2 | α |
| 1TQQ | 57.5 | 779.8 | 179.2 | β |
| 2B2F | 28.3 | 206.9 | 134.8 | α |
| 2RCR | 157.5 | 226.9 | 171.9 | α |
| 2D57 | 73.7 | 179.9 | 176.6 | α |
| 2FCP | 50.6 | 397.7 | 115.1 | β |
| 2K9Y | 48.6 | 68.3 | 128.1 | α |
| 2OCC | 110.9 | 1281.5 | 103.4 | α |
| 2RH1 | 100.4 | 958.0 | 178.6 | α |
| 2RLF | 40.5 | 183.3 | 175.7 | α |
| 3C9J | 2.8 | 27.6 | 52.8 | α |
| 2V8N | 55.2 | 329.8 | 165 | α |
| 3A2S | 35.0 | 285.1 | 151.9 | β |
| 3BRY | 28.0 | 102.7 | 136.9 | β |
| 3CSN | 17.4 | 281.6 | 150.5 | β |
| 3D4S | 84.6 | 1015.4 | 170.5 | α |
| 3EFM | 42.0 | 250.2 | 131.4 | β |
| 3EMN | 18.8 | 86.1 | 63.9 | β |
| 3LBW | 6.6 | 78.3 | 10.0 | α |
| 3LDE | 6.6 | 201.1 | 2.7 | α |
| 3MKT | 76.7 | 499.5 | 157.3 | α |
| 3QF4 | 95.7 | 2754.2 | 2.3 | α |
| 3V3C | 95.7 | 2754.2 | 2.3 | α |
| 3VVO | 55.3 | 505.4 | 136.9 | α |
| 4A01 | 73.6 | 2342.9 | 134.9 | α |
| 4APS | 20.9 | 273.9 | 168.8 | α |
| 4AQ5 | 58.3 | 2437.0 | 119.2 | α |
| 4DCB | 16.7 | 320.5 | 69.5 | β |
| 4EPA | 40.2 | 159.2 | 130.4 | β |
| 4IL3 | 22.4 | 721.1 | 15.5 | α |
