# Supplementary material for: A Model of Protein Association Based on Their Hydrophobic and Electric Interactions
Source: PLoS One. 2014 Oct 17;9(10):e110352. doi: 10.1371/journal.pone.0110352 (PMC4201486; doi:10.1371/journal.pone.0110352)
Supplement: References S1 — (DOCX) [file pone.0110352.s013.docx]

**SUPPLEMENTARY REFERENCES**

S1. Kim D, Park J, Kim SJ, Soh YM, Kim HM, Oh BH, Song JJ. (2013) Brucella Immunogenic BP26 Forms a Channel-like Structure. J Mol Biol 425: 1119–1126.

S2. Antonyuk S, Han C, Eady RR, Hasnain SS. (2013) Structures of protein–protein complexes involved in electron transfer. Nature 496: 123–127.

S3. Chandler P, Halbig KM, Miller JK, Fields CJ, Bonner HKS, Grabner GK, Switzer RL, Smith JL. (2005) Structure of the Nucleotide Complex of PyrR, the pyr Attenuation Protein from Bacillus caldolyticus. Suggests Dual Regulation by Pyrimidine and Purine Nucleotides. J Bacteriol 1773–1782.

S4. Banerjee S, Schmidt T, Fang J, Stanley CA, Smith TJ. (2003) Structural Studies on ADP Activation of Mammalian Glutamate Dehydrogenase and the Evolution of Regulation. Biochemistry 42: 3446–3456.

S5. Huard DJE, Kane KM, Tezcan FA. (2013) Re-engineering protein interfaces yields copper-inducible ferritin cage assembly. Nat Chem Biol 9: 169–176.

S6. Han S, Eltis LD, Timmis KN, Muchmore SW, Bolin JT. (1995) Crystal Structure of the Biphenyl-Cleaving Extradiol Dioxygenase from a PCB-Degrading Peudomonad. Science 270: 976–980.

S7. Smaoui M, Poitevin F, Delarue M, Koehl P, Orland H, Waldispühl J. (2013) Computational Assembly of Polymorphic Amyloid Fibrils Reveals Stable Aggregates. Biophys J 104: 683-693.

S8. Asenjo A, Chaterjee C, Tan D, Depaoli V, Rice W, Diaz-AvalosR, Silvestry M, Sosa H. (2013) Structural model for tubulin recognition and deformation by kinesin-13 microtubule depolymerases. Cell Rep. 3:759-768.
